# Multiscale Cell–Cell Interactive Spatial Transcriptomics Analysis

**DOI:** 10.1002/advs.202508358

**Published:** 2025-09-15

**Authors:** Sean Cottrell, Guo‐Wei Wei

**Affiliations:** ^1^ Department of Mathematics Michigan State University East Lansing MI 48824 USA; ^2^ Department of Computational Mathematics, Science, and Engineering Michigan State University East Lansing MI 48824 USA; ^3^ Department of Electrical and Computer Engineering Michigan State University East Lansing MI 48824 USA; ^4^ Department of Biochemistry and Molecular Biology Michigan State University East Lansing MI 48824 USA

**Keywords:** deep learning, multiscale cell–cell interactions, persistent Laplacian, spatial transcriptomics

## Abstract

Spatial transcriptomics data analysis integrates gene expression profiles with their corresponding spatial locations to identify spatial domains, infer cell‐type dynamics, and detect gene expression patterns within tissues. However, the current spatial transcriptomics analysis neglects the multiscale cell–cell interactions that are crucial in biology. To fill this gap, multiscale cell–cell interactive spatial transcriptomics (MCIST) analysis is proposed. MCIST combines the advantages of an ensemble of multiscale topological representations of cell–cell interactions in the gene expression space with those of cutting edge spatial deep learning techniques. MCIST is validated by a comparison of 14 cutting‐edge methods on a huge collection of 37 benchmark spatial transcriptomics datasets. It is demonstrated that MCIST yields superior performance in spatial domain detection. It achieves the best clustering score on 23/37 datasets and is among the top three methods on 33/37 datasets, while the second best method scored only 6/37 and 17/37 on these measures, respectively. In terms of overall performance with respect to a quantitative metric, MCIST offers more than an 11% improvement to the previous state‐of‐the‐art in spatial domain detection. In addition, MCIST offers multiscale insights with respect to trajectory inference, differentially expressed gene detection, and signaling pathway enrichment analysis. This MCIST sheds valuable light on the multiscale perspective in spatial transcriptomics.

## Graphical abstract

In this study, we present the MultiScale Cell‐Cell Interactive Spatial Transcriptomics Analysis method, which unites the strengths of spatially resolved deep learning techniques with a topological representation of multi‐scale cell‐cell similarity relations. The alignment of these methods induces a cell interaction feature set for enhanced spatial domain detection, differential gene expression analysis, and pseudotime inference.

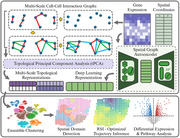

## 1 Introduction

The analysis of gene expression data plays a pivotal role in biological and medical research.^[^ ^1^ ^]^ In recent years, spatial transcriptomics has emerged as a powerful tool in advancing our understanding of the relationship between the gene expression of cells and their spatial distributions, which underpins tissue pathology and function. The applications of this spatial information are enormous, ranging from inferring cell–cell communication, trajectory inference, spatially variable gene detection, to spatial domain detection.^[^ ^2^, ^3^ ^]^

Among the clustering techniques developed for single‐cell RNA sequencing analysis, Louvain^[^ ^4^ ^]^ and Leiden^[^ ^5^ ^]^ incorporate only gene expression information into their workflows but neglect the crucial spatial dependency of gene expression. A variety of cutting‐edge spatial methods have been reported in the past few years, including SpaGCN,^[^ ^6^ ^]^ BayesSpace,^[^ ^7^ ^]^ SCAN‐IT,^[^ ^8^ ^]^ STAGATE,^[^ ^9^ ^]^ SpaceFlow,^[^ ^10^ ^]^ conST,^[^ ^11^ ^]^ BASS,^[^ ^12^ ^]^ stLearn,^[^ ^13^ ^]^ CCST,^[^ ^14^ ^]^ GraphST,^[^ ^15^ ^]^ PearlST,^[^ ^16^ ^]^ MuCoST,^[^ ^17^ ^]^ and SEDR.^[^ ^18^ ^]^ Among them, BayesSpace is a Bayesian statistical method that uses information from spatial neighborhoods for resolution enhancement of spatial transcriptomic data and for clustering analysis.^[^ ^7^ ^]^ SpatialPCA builds upon traditional probabilistic principal component analysis (PCA) to use a spot's spatial neighbors to infer latent factors, preserving the spatial correlation structure.^[^ ^19^ ^]^ stLearn normalizes gene expression using morphological similarity and utilizes the spatial information of these clusters to identify subclasses within the organization.^[^ ^13^ ^]^ PearlST employs a contrastive learning paradigm to extract histology informed features, then integrates a PDE‐based diffusion model to enhance characterization of spatial features at domain boundaries, and learns the latent low‐dimensional embeddings via Wasserstein adversarial regularized graph autoencoders.^[^ ^16^ ^]^ MuCoST employs dual expression similarity and spatial adjacency graph encoders to learn a shared latent embedding reflecting the multi‐view cell relations.^[^ ^17^ ^]^ SpaGCN^[^ ^6^ ^]^ is a spatial clustering method that uses graph convolutional networks (GCNs) to integrate gene expression, spatial location, and histological information to group spots into different spatial domains using unsupervised iterative clustering.^[^ ^6^ ^]^ SEDR is an unsupervised spatial clustering algorithm that utilizes a deep autoencoder to construct a low‐dimensional latent representation of gene expression data. This representation is then integrated with the corresponding spatial information using a variational graph autoencoder, allowing simultaneous spatial embedding during the clustering process.^[^ ^18^ ^]^ The conST method is a powerful and flexible spatial transcriptomics data analysis framework that uses contrastive learning techniques to learn low‐dimensional embeddings.^[^ ^11^ ^]^ Meanwhile, CCST clusters spatial transcriptomic data with a graph neural network, combining the gene expression and spatial information of single cells from spatial gene expression data.^[^ ^14^ ^]^ Local cell–cell interactions were taken into account in cell chats.^[^ ^20^ ^]^ A benchmark of various spatial methods has been reported.^[^ ^21^ ^]^ However, none of these methods provides a multiscale analysis of spatial transcriptomic data.

It is well established that cell–cell communication is inherently a multiscale process, including not only short‐range contact communication but also intermediate‐range,^[^ ^22^ ^]^ as well as long‐range interactions.^[^ ^23^ ^]^ Understanding this process requires integrating information on different scales to gain more comprehensive insight into cell domains, dynamics, function, and gene expression pathways. Despite significant progress in incorporating spatial information, current spatial transcriptomic analysis methods neglect these multiscale cell–cell interactions, which not only limits their performance but also represents a missed opportunity.

Gene expression data exhibits intricate geometric and topological structures, induced by cell–cell interactions in the expression space, prompting exploration into the biological significance of these structures. The Riemannian curvature of low‐dimensional manifolds in scRNA‐seq data has been analyzed for single‐cell RNA‐seq data.^[^ ^24^ ^]^ Differential geometry learning, through Gaussian and mean curvatures of cell‐cell interactive manifolds, has been introduced for cell‐type classification.^[^ ^25^ ^]^ Hyperbolic geometry has also been applied to model cell differentiation trajectories.^[^ ^26^ ^]^ Furthermore, single‐cell RNA velocity fields have been analyzed using Hodge decomposition to reveal cell dynamics.^[^ ^27^ ^]^ Algebraic topology has emerged as a popular tool in gene expression data analysis, giving rise to single‐cell topological data analysis (scTDA).^[^ ^28^ ^]^ Spectral simplicial theory has also been utilized to analyze genomic data.^[^ ^29^ ^]^ The topological PCA method was introduced to capture multiscale topological structures in RNA sequencing data.^[^ ^30^, ^31^ ^]^ Ricci curvature has been employed to analyze cell states in single‐cell transcriptomic data.^[^ ^32^ ^]^ However, substantial biological insight has been gained from geometric and topological analysis, intrinsic multiscale cell–cell interactions in spatial transcriptomics have not yet been studied.

The objective of the present work is to introduce multiscale cell‐cell interactive spatial transcriptomics (MCIST) analysis. We construct an ensemble of topological PCA embeddings at various scales to capture multiscale cell‐cell interactions in gene expression data. This ensemble of low‐dimensional multiscale gene expression representations is paired with a cutting‐edge spatial representation extracted from the latent space of a deep learning model for cell clustering. We also develop a novel unsupervised optimization technique to construct an effective low‐dimensional gene expression representation for downstream analysis, such as trajectory inference and differential expression analysis. We validate MCIST analysis on a large collection of 37 spatial transcriptomics datasets acquired using diverse sequencing and imaging‐based technologies. Extensive comparisons with a wide variety of other established methods from the literature demonstrate that MCIST is the state‐of‐the‐art technique, which not only provides a new standard for the field but also sheds light on the multiscale perspective in spatial transcriptomics.

## 2 Results

### 2.1 Overview of MCIST

**Figure 1:**
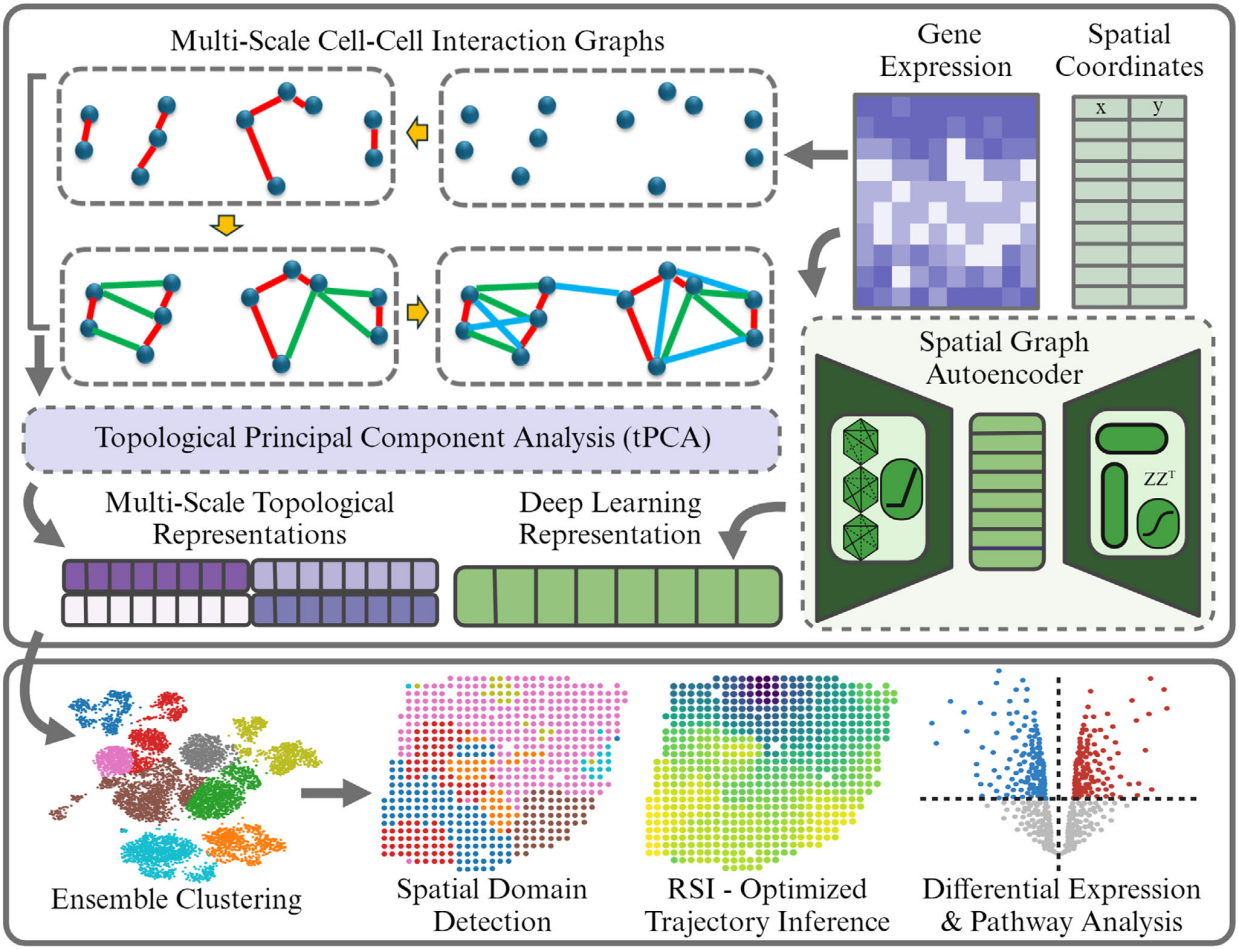
Overview of MCIST workflow. Gene expression data are treated as a point cloud of cells, from which we construct a sequence of multiscale cell‐cell interaction graphs based on an affinity measure between expression profiles and k‐nearest neighbors (kNNs). These graphs give rise to an ensemble of low‐dimensional multiscale topological PCA representations of the gene expression data, each characterizing a specific combination of cell–cell connectivities. A latent space representation of the spatially resolved gene expression data is also constructed from a deep learning model to pair with the multiscale topological representation. These representations are then aligned for downstream ensemble clustering‐enabling spatial domain detection, residue‐similarity index (RSI)‐optimized trajectory inference, and differential gene expression analysis.

MCIST is a two‐component protocol tailored to extract the multiscale cell‐cell interactions in the gene expression space along with the spatial information inherent in the data as shown in **Figure** **1**. First, a model of cell–cell interactions is characterized by the multiscale correlations among their gene expression profiles. Specifically, we construct a sequence of cell–cell interactive graphs induced by varying multiple k‐nearest neighboring (kNNs) where the edges are weighted by the Gaussian kernel affinity measure. We then seek to preserve these cell–cell interactions during dimensionality reduction by combining a spectral graph regularization technique with traditional nonlinear sparse PCA, but with multiscale topological Laplacians in place of the traditional graph Laplacian, rendering an ensemble of multiscale 'topological PCA' representations.

To incorporate the tissue's spatial dependencies, we adopted a spatially informed deep learning technique to generate an additional latent representation of the data and pair it with the multiscale topological representations. Among various spatially informed deep learning methods, STAGATE is the most scalable in terms of runtime and memory usage^[^ ^9^ ^]^ according to the latest benchmarking study.^[^ ^21^ ^]^ However, it was also found to be less competitive than several more sophisticated and computationally intense approaches, such as GraphST or Spaceflow.^[^ ^21^ ^]^ Therefore, there are two routes that one could take with MCIST‐ either prioritizing performance by pairing with a more powerful model such as GraphST^[^ ^15^ ^]^ and Spaceflow,^[^ ^10^ ^]^ or prioritizing efficiency by resorting to a method such as STAGATE. In this study, we thoroughly investigate the performances of both approaches.

The multiscale cell–cell interactive representations and the spatial deep learning representation are utilized in agglomerative clustering, enabling us to detect spatial domains and then analyze differential gene expression patterns and enriched pathways in each segmented region of the tissue. For trajectory inference, we can extract the ‘best’ embedding according to an unsupervised optimization procedure. Specifically, we choose the single set of topological PCA features that maximize the Residue‐Similarity Index (RSI) score^[^ ^33^ ^]^ for diffusion pseudotime analysis. In some cases, such as on the STARmap data, we may even improve clustering performance by identifying the spatial domains based on the RSI‐inferred gene expression embedding alone, rather than an ensemble approach. A more in‐depth look at this ensemble and optimization approach is seen in Figures S3 and S4 (Supporting Information).

### 2.2 Overview of Datasets

To validate the proposed MCIST, we have collected 37 datasets, which represent the largest collection of spatial transcriptomics datasets to our best knowledge in such a study. We provide a concise summary of these datasets in Table S1 (Supporting Information). These datasets were obtained from a variety of spatial technologies and vary dramatically in the number of samples (spots/cells) ranging from 176 to 18 670, in dimensions (genes) from 79 to 33 538, and in the number of clusters from 4 to 18. As such, they pose a severe challenge to all existing methods and serve as benchmark datasets for new spatial methods in the field. A more thorough description of the datasets and their summary statistics can be found in Supporting Information (Tables S2– S7). For fairness, the results for other methods on the Visium, MERFISH, BaristaSeq, StereoSeq, and STARmap data were obtained from a recent benchmarking study of spatial clustering techniques.^[^ ^21^ ^]^ The results on the ST data were obtained using the benchmarking study's reproducible pipeline and averaged over calculations with 10 random seeds. All reported MCIST results in this study are averaged over calculations with 50 random seeds.

### 2.3 Evaluating MCIST on Benchmark Visium Dorsolateral Prefrontal Cortex Data

**Figure 2:**
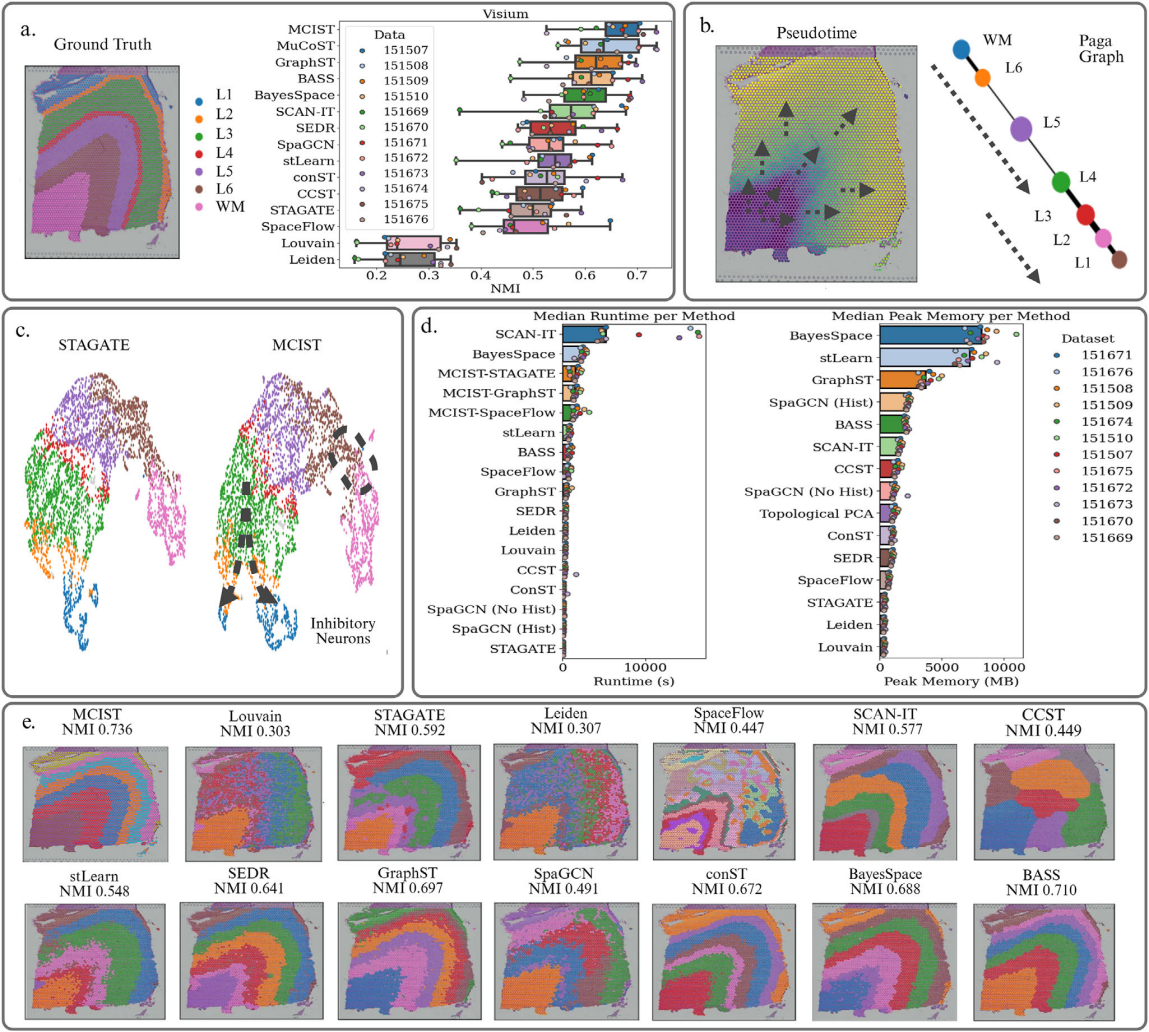
a) Ground truth annotations of Slice 151673 and a performance comparison across different leading methods. Sample 151673 labels correspond to White Matter and neuronal layers 1 through 6. The bar chart depicts the comparison of NMI scores averaged over all 12 DLPFC datasets between MCIST and other cutting‐edge methods. Results indicate that MCIST is the new state‐of‐the‐art. For fairness, results for other methods were adopted from the latest published benchmarking study on spatial clustering.^[^ ^21^ ^]^ Methods are ranked according to average performance, with MCIST in the lead. b) Pseudotime analysis of Slice 151673 accurately reflects the inside out pattern of neurogenesis. Pseudotime values increase from the white matter region in a smooth, vertical fashion outwards, reflecting the trend for the youngest neurons to migrate to the outermost layers. The Paga graph also demonstrates this pattern, with the clusters forming a straight line from the WM cluster to the outer neuronal layers. c) Comparison of UMAP embeddings between the STAGATE and MCIST (when combined with STAGATE) latent features. We note that the addition of cell–cell interaction information effectively enables the detection of what may be the branching inhibitory/excitatory neuron development trajectories in Layers 2/3. Also, the transition state between the WM and L6 neurons (subplate zone) is represented as a slightly thinner bottleneck region, better emphasizing the negative graph curvature of the transition zone ^[^ ^32^ ^]^ d) Comparisons of average run‐time and memory usage for multiple methods adopted from the latest benchmarking study along with different MCIST variants.^[^ ^21^ ^]^ e) Comparison of spatial domain detection on Slice 151673 between MCIST and other methods. The MCIST spatial domains most accurately reflect the ground truth annotations visually. For fairness, plots of other methods are obtained using the pipeline made available by the latest benchmarking study.^[^ ^21^ ^]^

We first quantitatively compare the performance of MCIST with fourteen other top‐performing methods for spatial domain detection. To this end, we benchmark our technique on the LIBD dorsolateral prefrontal cortex data (see **Figure** **2**a), which contains the spatially resolved transcriptomic profiles of 12 slices as well as their manual annotations of neuronal layers and white matter.^[^ ^34^ ^]^ As shown in Table S1 (Supporting Information), this data has a very high dimension (33 538), which can be challenging for many methods.

After performing ensemble clustering with various connectivity weighting combinations in topological PCA and combining it with the latent embeddings of a spatial deep learning method, specifically GraphST, MCIST was able to achieve a mean normalized mutual information (NMI) score of 0.660 across all 12 datasets. This improved the performance of the baseline GraphST model by nearly 8% according to the most recent spatial clustering benchmarking results, where the results of each method are averaged over 10 predictions.^[^ ^21^ ^]^ Additionally, we include results from the more recently developed MuCoST method, which philosophically shares some similarities with MCIST given their inclusion of transcription similarity based graph encoders.^[^ ^17^ ^]^ Reproducing their results via their publicly shared pipeline, we observe that MCIST manages to offer a 2.6% improvement over this method as well. We showcase these results in Figure 2a. GraphST was selected to maximize our performance. Alternatively, we might combine with a graph attention autoencoder (STAGATE) to take the advantage of its short runtime, low memory, and scalability as shown in Figure 2d. We observe that MCIST paired with STAGATE still achieves a competitive scalability performance relative to the other spatial methods, particularly BayesSpace and SCAN‐IT. Similarly, when paired with GraphST or SpaceFlow the model still manages to offer competitive runtimes. In terms of memory usage, when performed on all 12 datasets the inclusion of MultiScale Topological PCA modeling increased the maximum memory allocation by roughly 1000MB on average. Meanwhile, the inclusion of multiscale cell–cell interaction information improved the performance of STAGATE by 31.35% relative to the reported results in the benchmarking study, and still managed to achieve state‐of‐the‐art results by a margin of more than 5%, further validating the proposed strategy. A complete account of the results for MCIST and other methods can be found in Table S8 (Supporting Information).

Given the cluster assignments and MCIST latent features, we can also perform trajectory inference to gain insights into the biological dynamics taking place in this tissue, particularly with regard to neuronal development. In Figure 2b, we used the PAGA trajectory inference tool in Scanpy^[^ ^35^ ^]^ to calculate diffusion pseudotime values from the MCIST latent embeddings. Choosing a white matter (innermost cluster) spot as our root node, we observe the pseudotime values increasing smoothly in a vertical fashion outward toward the outskirts of the cortex. This pattern reflects a known biological process in neuro‐development called the “inside‐out” pattern of corticogenesis. Newly born neurons form in the ventricular zone and migrate through the intermediate zone along radial glia fibers to the cortical plate, which develops between the marginal zone and subplate. The cortical plate forms inside out, such that the youngest neurons migrate to the outer most layers.^[^ ^36^ ^]^

This information is further captured via the construction of a PAGA graph.^[^ ^35^ ^]^ We observe sequential connections between the inner cortical layers leading to the outer layers, accurately reflecting the expected temporal ordering. Therefore, we note that the MCIST embeddings are also capable of deriving important biological information via downstream analysis after initial tissue‐type identification and spatial domain reconstruction. In Figure S13 (Supporting Information), we examine the superiority of MCIST embeddings compared to some other popular methods, such as Scanpy and stLearn, in its ability to extract these insights. In Figure 2c, we display the UMAP embeddings produced from the STAGATE and MCIST (paired with STAGATE) representations. We note a few distinct differences between the two which highlight the important biological insights gained from considering cell‐cell interactions. First, recent studies have identified transition cell states as forming thin bridges between stable communities or states in PCA and UMAP embeddings, or rather, areas of negative graph curvature.^[^ ^32^ ^]^ We see that the region between the White Matter and Layer 6 neurons‐ or, roughly speaking, the subplate zone, forms such a bridge in both visuals. However, we observe that it is slightly thinner in the MCIST UMAP embedding. The subplate zone is crucial for the transition of developing neurons and axonal pathways, and is a key transitional area in cortical development. We may argue that the MCIST visual does a better job of emphasizing this point with its thinner bridge, as fewer overlapping samples between the stable communities would result in a more strongly negative graph curvature in this region, highlighting its relevance. More strikingly, we observe a clearer branching in the L2/3 layers in the MCIST embedding than in the STAGATE embedding. This may correspond to the divergence into excitatory and inhibitory neuronal cell types observed in the upper layers by Maynard et al.^[^ ^34^ ^]^ We conclude that there is a more extensive biological understanding to be gained from downstream analysis with the MCIST embeddings than just the STAGATE embeddings.

In Figure 2e, we select one slice (#151673) for further analysis. We note that default Scanpy methods, such as Leiden and Louvain clustering, perform the worst, given that they do not incorporate any spatial information. Specifically, we note that both methods were only able to accurately detect the white matter regions, with each of the other six clusters being mixed among the neuronal layers. SpaGCN, STAGATE, and stLearn produced clusterings that were closer in their structure to the ground truth, but without clear and clean separations between the neuronal layers. SEDR and GraphST, two state‐of‐the‐art spatially informed deep learning techniques, were both able to fairly accurately reconstruct the overall architecture of this slice, but were both edged out in performance by MCIST, which offers the best separations of the layers.

Figure 2d shows the runtime and memory comparisons of some spatial clustering methods. Among the deep learning‐based methods, STAGATE has the lowest runtime and memory usage, which motivates us to pair our framework with STAGATE. However, in cases where STAGATE cannot achieve reasonable performance, we can instead pair our multiscale gene representations with more computationally expensive methods, such as GraphST or SpaceFlow.

### 2.4 Evaluating MCIST on Cancer Progression in Spatial Transcriptomics HER2 Positive Breast Tumor Data

**Figure 3:**
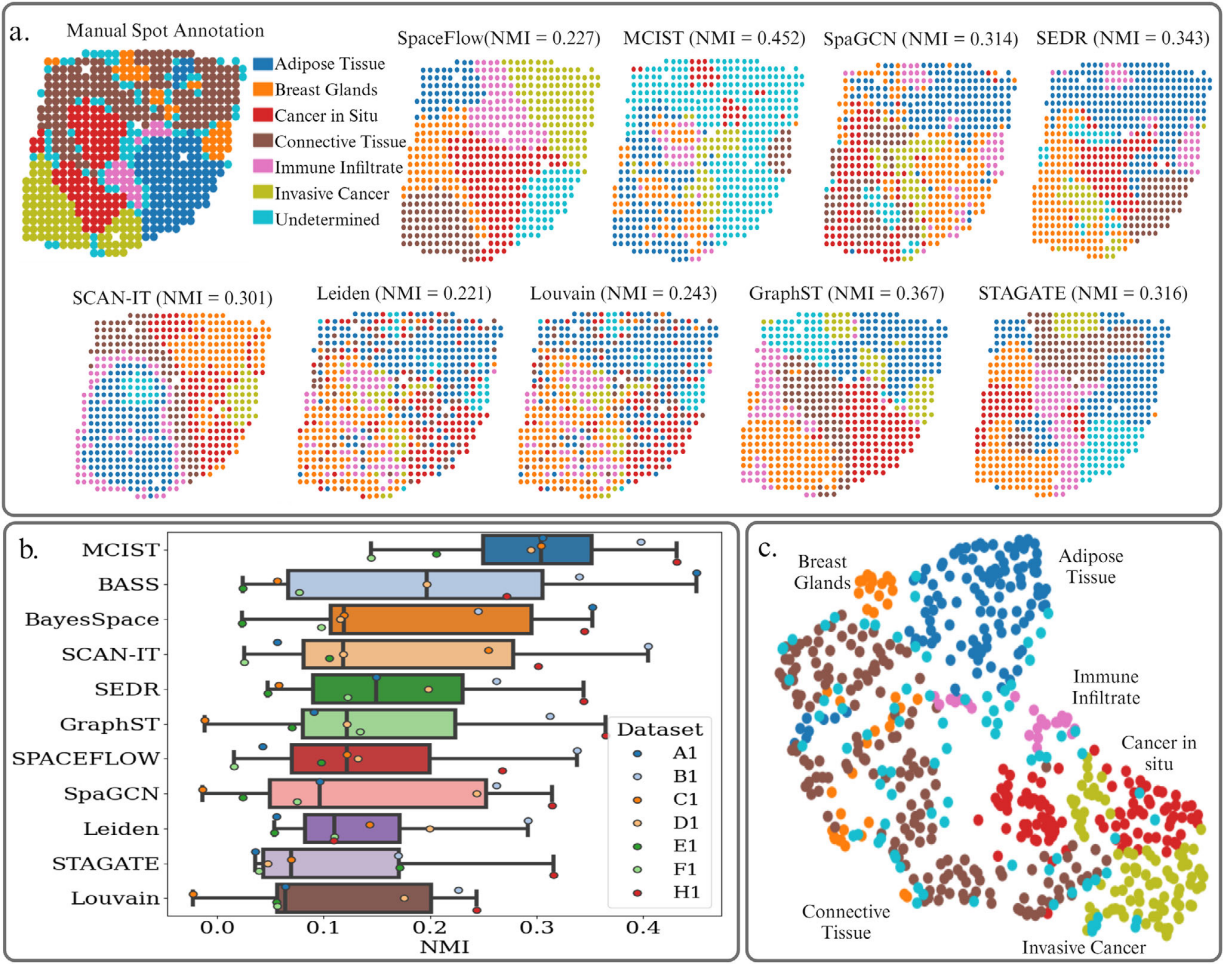
a) Ground truth annotations of dataset H1 from the HER2 Positive Breast Tumor data. Spots are annotated as adipose tissue, breast glands, immune infiltrate, invasive, and noninvasive cancer. We also compare several spatial domain detections on this sample between MCIST (paired with GraphST) and other tested methods. MCIST spatial domains provide a more thorough segmentation of the diseased tissue region while the overall accuracy measure (NMI) is also the best. b) Comparison of NMI scores over all 7 datasets between MCIST (paired with SpaceFlow) and other leading methods. Results indicate that MCIST is able to significantly outperform other state‐of‐art methods for spatial domain detection. The results for the other methods were computed using the pipeline made available by the latest benchmarking study.^[^ ^21^ ^]^ Methods are ranked according to average performance. c) UMAP visualization of the H1 sample produced from the MCIST (paired with STAGATE) embedding shows a discernible separation between the healthy and diseased tissue types.

It is estimated that about one in eight US women will develop invasive breast cancer over the course of their lifetime. As such, gaining a more thorough understanding of the biological pathways behind cancer development is a primary research goal.^[^ ^37^ ^]^ Here, we analyze the HER2‐positive breast tumor data, a collection of seven datasets that were manually annotated by pathologists as either various healthy tissue regions, noninvasive cancer, or invasive cancer as shown in **Figure** **3**a. As shown in Table S1 (Supporting Information), these datasets have relatively small numbers of samples, which may not favor deep learning methods. In Figure 3b, it is seen that MCIST (paired with SpaceFlow) achieved a mean NMI score of 0.298, outperforming the second‐best method, BASS, by 47% in all seven datasets. Another top performer is BayesSpace. Both BASS and BayesSpace are probability based methods, rather than deep learning. This would support the notion that datasets with such a small number of samples do not favor spatial deep learning techniques.

A case study is presented in Figure 3a, where we visually compare the spatial domains of nine of the tested methods on the H1 dataset, which contained 613 spots and 15 029 genes. For this case study, we combine MCIST with GraphST rather than SpaceFlow to illustrate the versatility of the method in deriving different biological insights by utilizing different deep learning techniques. Again, we note that the baseline methods Louvain and Leiden, which are not spatially informed, perform the worst. Although Louvain manages to somewhat accurately detect the cancerous region in the lower‐left section of the tissue, the connective tissue region and breast glands region toward the top, and the adipose tissue region on the right side of the tissue, it fails to cleanly separate the clusters, leading to very low measures of accuracy according to NMI. Spatially informed methods such as GraphST and STAGATE manage to fairly accurately separate the pre‐cancerous from the cancerous tumor regions but cannot reliably detect either the healthy breast gland regions or immune infiltrate, both of which are fundamental for understanding various aspects of the tumor environment and cancer progression.^[^ ^37^, ^38^ ^]^ SEDR and SpaGCN, meanwhile, perform poorly in faithfully clustering the tumor regions. Equipped with GraphST, the MCIST spatial domains, however, achieve the highest measure of accuracy and are the best in meaningfully segmenting the tumor and immune micro‐environment. However, we note that the MCIST clustering was unable to distinguish between the adipose connective tissue and the broader connective tissue region. However, we might reasonably argue that a detailed partition of the tumor and immune environment is a more significant development than distinguishing between these two types of healthy connective tissue in this context. In terms of NMI, MCIST outperforms GraphST by a wide margin of 23%. GraphST was also the second best performing method in this sample. We again note the results in Figure 3b in all 7 datasets, where MCIST (combined with SpaceFlow) improved the performance of the baseline deep learning method by up to 105% on average. For efficiency, we may also combine with STAGATE, in this case MCIST is still able to outperform the second‐best BASS by 17.8% on average, which is a significant margin of improvement. In Figure 3c, we show the UMAP visualization of the MCIST embeddings when paired with STAGATE, and note that it is able to provide a reasonable separation between the healthy and diseased tissue regions. A complete account of the results for MCIST and other methods can be found in Table S9 (Supporting Information).

**Figure 4:**
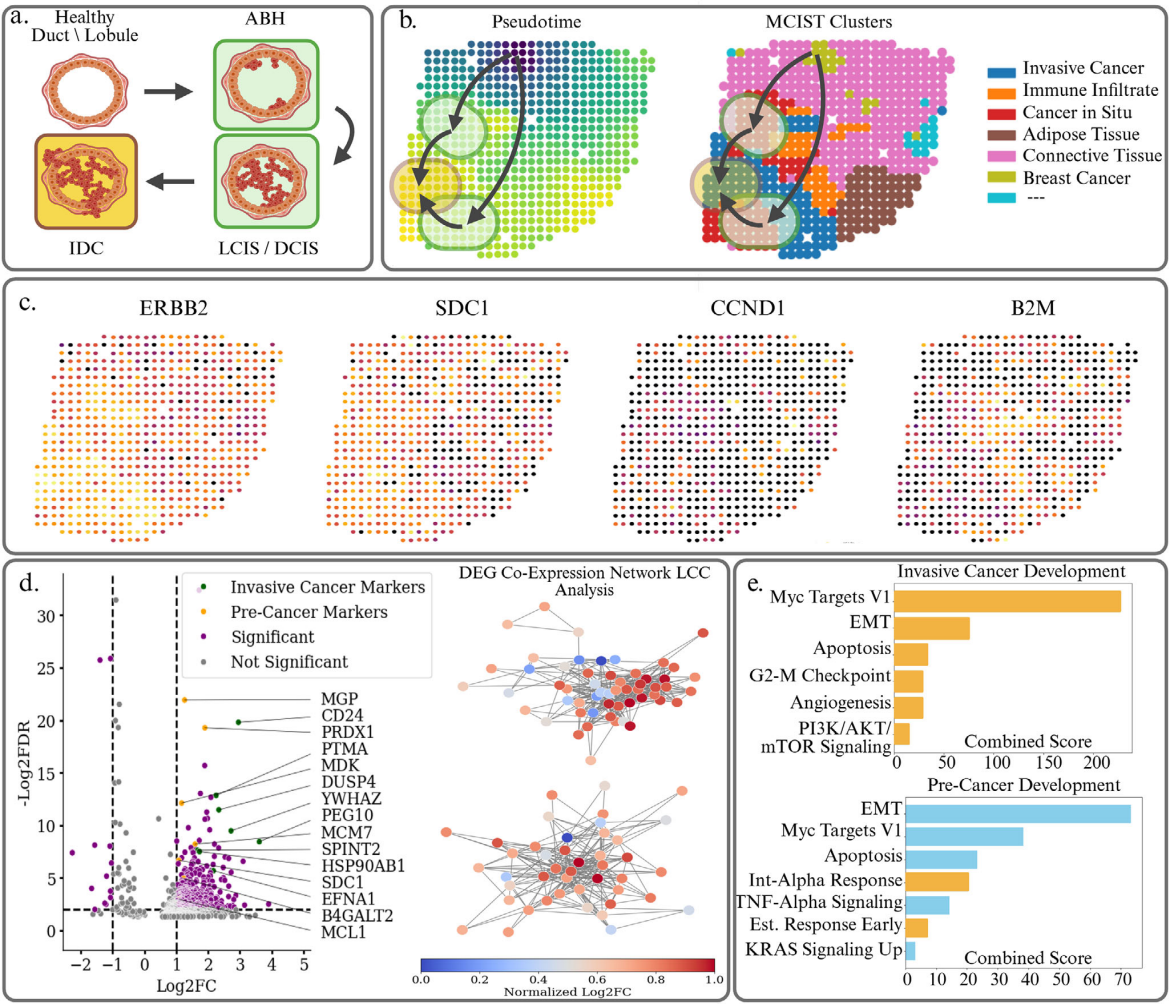
a) Typical development of breast cancer from healthy epithelial cells to atypical breast hyperplasia (ABH), ductal carcinoma in situ (DCIS) or lobular carcinoma in situ (LCIS), and to invasive ductal carcinoma (IDC). b) Pseudotime analysis on the MCIST and STAGATE latent features of dataset H1 depicts this development from healthy breast glands to noninvasive to invasive cancer. We observe the healthy breast gland spots surrounding the root spot have the lowest pseudotime values, while the cancer in situ domains has a slightly higher pseudotime value, and the invasive cancer spots have the highest pseudotime values, which accurately reflects the typical development of breast cancer. c) Spatial expression map of various pathology inducing or related genes including ERBB2, SDC1, CCND1, and B2M. d) Differential gene expression analysis between healthy tissue and pre cancerous regions as well as pre cancer and invasive cancer regions detected by MCIST (paired with STAGATE). Some specific genes contained within the largest connected components of gene co‐expression networks are highlighted as they correspond well to biologically meaningful markers which contribute to the development of cancer and the increase of tumor aggression. e) Pathway enrichment analysis between healthy breast glands and noninvasive cancer, as well as noninvasive and invasive cancer regions identifies significant biological processes that were not detected as varying among the STAGATE spatial domains, indicating that the additional cell interaction information benefits downstream biological analysis. Pathways which were identified among the MCIST spatial domains but not the STAGATE domains are highlighted in yellow.

The leading hypothesis for the development of breast cancer is the stepwise progression from healthy epithelial cells lining the ducts and lobules of the breast glands to atypical breast hyperplasia, ductal/lobular carcinoma in situ (DCIS/LCIS), a noninvasive pre‐cancer condition, and to invasive ductal carcinoma (IDC).^[^ ^37^ ^]^ There is consensus that DCIS/LCIS will eventually develop into invasive cancer, or IDC, in the absence of intervention as shown in the diagram in **Figure** **4**a. While breast cancer is certainly a heterogeneous disease, approximately 90% of diagnoses fall under invasive ductal or lobular carcinoma. Understanding the dynamics behind the development of these cancers and the biological pathways driving them is therefore a critical step in predicting their behavior and determining the most effective treatments, including patient‐specific medicine. To do this, we performed trajectory inference analysis on a MCIST (paired with STAGATE) latent embedding using diffusion pseudotime, taking a healthy breast gland spot as our root node (Figure 4b). We observe that the pseudotime values accurately reflect this expected trajectory, with values increasing along the clusters corresponding to noninvasive and invasive tumor regions among the MCIST (paired with STAGATE) spatial domains. This indicates that the MCIST latent embeddings of gene expression profiles are able to accurately encode the dynamic relations between tissue types in the tumor microenvironment. This progression aligns well with the spatial distribution of known disease‐associated genes. For example, ERBB2 expresses most strongly in the MCIST clusters corresponding to invasive cancer, which is also the highest pseudotime region. This indicates that these cells have fully activated HER2 signalling, which is consistent with the in‐situ to IDC transition. SDC1 is expressed most strongly in the cancer‐in‐situ regions in the MCIST plot, corresponding also to increased pseudotime positioning just before the highest values where full invasiveness has begun. Syndecan‐1 is up‐regulated on ductal epithelium undergoing EMT‐like changes and matrix remodeling. This pattern therefore matches the in‐situ compartment transitioning toward invasiveness.^[^ ^39^ ^]^ CCND1 shows increased expression in both the in‐situ and invasive regions, and is concentrated mostly in the regions where pseudotime is increasing from green (middle values) to yellow (highest values). CCND1 marks the proliferation surge as ducts breach the basement membrane.^[^ ^40^ ^]^ Lastly, B2M is expressed highly in the immune infiltration regions and the surrounding tumor, which show earlier pseudotime values. B2M is an MHC‐I component enriched on antigen‐presenting immune cells and stromal elements recruited around emerging lesions. In Figure 4d, we then performed differential gene expression and pathway enrichment analysis on the spatial domains detected via MCIST using the gene set enrichment analysis python library (GSEApy). After collecting the differentially expressed genes of the non‐invasive and invasive cancer spatial domains, we constructed gene co‐expression networks for each cluster. We then extracted the largest connected components from these GCNs and analyzed the pathways regulated by these sets of genes.

We first consider differentially expressed genes between the healthy breast gland region and the non‐invasive tumor regions detected by MCIST. We used Scanpy to select the genes that were differentially expressed according to Welch's t‐test with a p‐value ⩽ 0.05. In Figure 4c, we display the differentially expressed genes from these spatial domains and note such results as the over‐expression of ERBB2 (HER2), which encodes a member of the epidermal growth factor receptors and would contribute to the development and progression of certain aggressive types of breast cancers. Also, MCM7 is among the known biomarkers for cancer development that are targets for therapies.^[^ ^41^ ^]^ Given the set of differentially expressed genes, we identify those belonging to the largest connected component of a gene co‐expression network, and then perform pathway enrichment analysis with the GSEApy to infer which biological processes are significantly varying between the healthy tissue and the pre‐cancer, as shown in Figure 4e. We utilized the MSigDB Hallmark 2020 gene set as a reference and selected statistically significant pathways with a p‐value ⩽ 0.05. In yellow, we identify the biologically and statistically significant pathways that were found to vary between the MCIST spatial domains but did not vary among the STAGATE spatial domains. Epithelial Mesenchymal transition is a hallmark of the development of breast cancer, as healthy epithelial cells lining the ducts and lobules of the breast glands acquire invasive properties. Aberrations in pathways such as KRAS signaling up, Myc Targets I, and TNF‐Alpha signaling can create a tumor‐promoting environment and lead to tumorigenesis.^[^ ^42^, ^43^, ^44^ ^]^ We may speculate that multiscale analysis based on transcription similarity paired with deep spatial learning encourages the formation of clusters that emerge along functional gradients to a greater extent than the single inclusion of spatial information would allow. This creates a more coherent DEG list among the domains, allowing us to extract more insightful pathways for analysis.

We also analyzed the differentially expressed genes among the invasive cancerous regions. We note significant upregulation of genes such as EFNA1, PTMA, SPINT2, and CD24, which have previously been indicated in increased tumor aggression and driving the transition to invasive forms of breast cancer.^[^ ^45^ ^]^ Angiogenesis signaling and PI3K‐Akt signaling, captured in Figure 4e, are pathways which would promote cell mobility through alterations in cell adhesion or through the formation of new blood vessels, and therefore influence the development of invasive properties.^[^ ^46^ ^]^ Apoptosis and G2‐M Checkpoint, meanwhile, affect the proper regulation of the cell cycle and programmed cell death. It is interesting to note that among these mentioned biologically significant pathways, only a few were identified as varying significantly between the noninvasive and invasive cancer regions from the STAGATE‐detected spatial domains (i.e., those in bright blue in Figure 4e). We can therefore conclude that the MCIST spatial domains lend themselves to considerably more insightful and thorough downstream analysis than those produced by the base deep learning method STAGATE. In Figure S12 (Supporting Information), we perform a similar analysis dissecting the heterogeneity in the tumor micro‐environment of a Visium Breast Tumor dataset, further validating this conclusion.

### 2.5 Evaluating MCIST on MERFISH and BaristaSeq Data

Aside from the 10x Visium and ST platforms, we investigated the generalization ability of MCIST for imaging‐based molecular data such as MERFISH, and optimized in situ barcode sequencing with BaristaSeq.^[^ ^47^, ^48^ ^]^ These datasets are both characterized by a relatively small number of genes, which may favor certain methods that work well for low dimensions. Ultimately, we found that cell–cell interaction regularization significantly improved performance on both the BaristaSeq and MERFISH data when combined with the embeddings from SpaceFlow.

MERFISH is a high‐resolution imaging technique that enables the simultaneous visualization and quantification of thousands of RNA molecules within individual cells, preserving their spatial context. We focused our analysis on a mouse hypothalamic preoptic region, which contains five annotated slices with 155 genes sequenced.^[^ ^47^ ^]^ The MERFISH data is characterized by enhanced spatial resolution and targeted detection of genes, with each dataset containing 155 genes. MERFISH's spatial resolution positions it as a gold standard for single‐molecule sensitivity and sub‐cellular spatial precision alongside technologies such as StereoSeq, seqFISH, Xenium, and CosMx.^[^ ^49^, ^50^ ^]^

**Figure 5:**
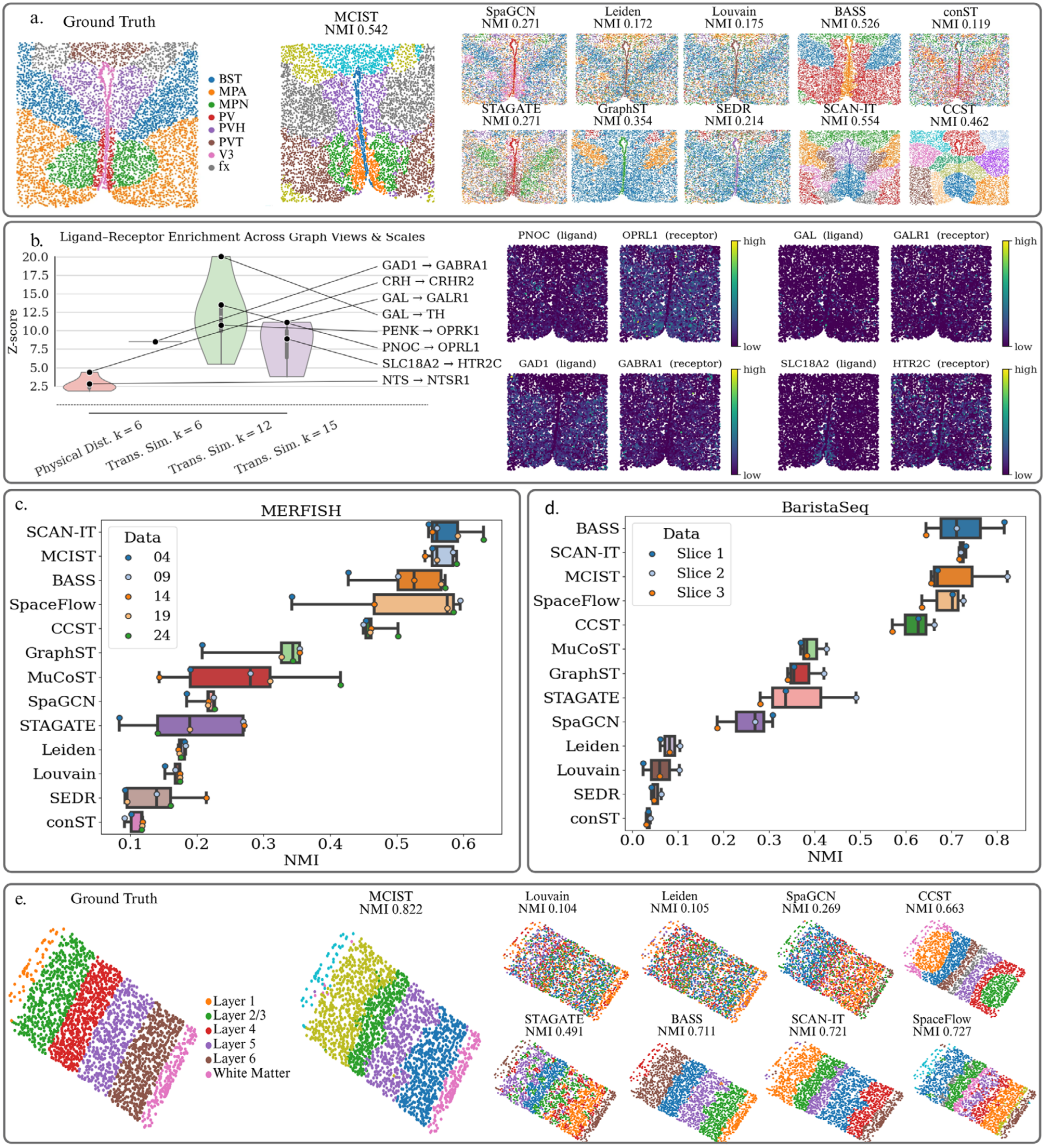
a) Ground truth annotations of MERFISH dataset 19 and comparisons of spatial domain detection between MCIST (paired with SpaceFlow) and other methods. Several other deep learning methods perform extremely poorly in detecting the MERFISH spatial domains while MCIST domains accurately reflect the true tissue structure. Results of other methods were computed using the pipeline made available by the latest benchmarking study.^[^ ^21^ ^]^ b) Distributions of ligand–receptor enrichment on the edges of various graph views and scales demonstrate that cell–cell interactions commonly manifest as both physical spatial adjacency as well as transcription similarities or correlations across multiple scales, motivating the modeling framework of MCIST. c) Comparison of NMI scores between MCIST (paired with SpaceFlow) and other methods over all five MERFISH datasets. MCIST is able to outperform all spatially informed deep learning techniques on average except for SCAN‐IT. Results of other methods were adopted from the latest benchmarking study.^[^ ^21^ ^]^ Methods are ranked according to average performance. d) Comparison of NMI scores between MCIST (paired with SpaceFlow) and other methods over all three BaristaSeq datasets. Results of other methods were adopted from the latest benchmarking study.^[^ ^21^ ^]^ Methods are ranked according to average performance. e) Ground truth annotations of BaristaSeq dataset Slice 2 and comparisons of spatial domain detection between MCIST and other methods. Results of other methods were computed using the pipeline made available by the latest benchmarking study.^[^ ^21^ ^]^

The results in **Figure** **5** demonstrate the versatility of multiscale cell‐cell interaction learning in enhancing the performance of spatially aware deep learning methods for spatial domain detection on a variety of different types of data. Specifically, in Figure 5a, we compare several methods for spatial domain detection on dataset 19 of the MERFISH data. The dataset comes from a mouse hypothalamic preoptic region and is comprised of 8 tissue types: BST (Bed Nucleus of the Stria Terminalis), MPA (Medial Preoptic Area), MPN (Medial Preoptic Nucleus), PV (Paraventricular Nucleus of the Hypothalamus), PVH (Paraventricular Hypothalamic Nucleus), PVT (Paraventricular Thalamic Nucleus), V3 (Third Ventricle), and fx (Fornix). We observe that SpaGCN, GraphST, STAGATE, and SEDR are mostly unable to clearly reconstruct the true tissue architecture, except for the V3 region. Likewise, the methods that do not account for the spatial context, Louvain and Leiden, perform very poorly. BASS^[^ ^12^ ^]^ and SCAN‐IT,^[^ ^8^ ^]^ meanwhile, both manage to detect the spatial domains fairly accurately, though neither seems to be able to reliably separate the MPA and MPN regions. MCIST, meanwhile, achieves a fairly accurate separation among all the tissue types. In Figure 5e, we perform a similar comparison, focusing on dataset two from the BaristaSeq data. Like the Visium DLPFC data, the slice is taken from a brain cortex region, separated to form fairly linear layers. We again note that the spatially unaware methods, Louvain and Leiden, are not able to discern any of the neuronal layers, and only manage to clearly identify the White Matter region. SpaGCN and STAGATE, meanwhile, are unable to cleanly separate the neuronal layers. BASS, SCAN‐IT, and MCIST all manage to fairly accurately reflect the ground truth.

In Figure 5c, we observe that spatially informed deep learning techniques, which performed well on the Visium and HER2 data, were found to perform very poorly on the MERFISH datasets, such as GraphST, SpaGCN, SEDR, and STAGATE. STAGATE, for example, achieved a mean NMI score of just 0.191 over all five datasets. Other methods, like SCAN‐IT and SpaceFlow, were able to perform quite well. MCIST was able to achieve a mean NMI score of 0.566 when paired with SpaceFlow, an improvement of 10.3% over the base deep learning model. Likewise, some spatially aware deep learning methods performed poorly on the BaristaSeq data, as seen in Figure 5d. BaristaSeq is a spatial technology designed to enhance the efficiency and accuracy of in situ barcode sequencing.^[^ ^48^ ^]^ The number of unique genes in the three BaristaSeq datasets is only 79. Over these three datasets, MCIST, paired with SpaceFlow, achieved a mean NMI of 0.715, which is about a 4% increase in performance over the reported SpaceFlow results.^[^ ^21^ ^]^ We may speculate that cell–cell interaction analysis of the gene expression data with such a small amount of genes may not be as beneficial in certain cases. Yet, as the sequencing depth continues to improve with new technologies, this issue will become increasingly irrelevant. A full accounting of the results for MCIST and other methods on both platforms can be found in Tables S10 and S13 (Supporting Information).

In Figure 5b we include an analysis of MERFISH sample 19 examining the physical spatial variance of activity of ligand receptor pairs versus the spatial variance of the activity of ligand receptor pairs on the various stages of expression similarity graph filtration. We observe a unique enrichment of pairs across the different graph configurations, suggesting the presence of cell interactions that are captured by spatial proximity as well as longer‐range transcriptional programs that correlate across distant regions. This underscores the multi‐view and multiscale nature of cell communication, which motivates the modeling framework of MCIST. We perform this experiment by examining the pairwise enrichment of a ligand receptor pair on the edges of each graph and comparing to a baseline random distribution of that same pair. The observed enrichment versus the null hypothesis enrichment gives us a Z‐Score from which we can infer how well that graph configuration captures the putative LR interaction. Specifically, we note that on the spatial graph we have strong Z‐Scores for such LR pairs as CRH‐CRHR2. CRH is secreted en passant by stress‐responsive interneurons; its type‐2 receptor is membrane‐bound on the same or immediately adjacent cells.^[^ ^51^ ^]^ The transcription similarity graph with *k* = 6 shows a high score for the GAD1‐GABRA1 pair. GAD1 encodes the GABA‐synthesising enzyme GAD67; GABRA1 encodes the principal α1 sub‐unit of the GABA‐A receptor. Cells that are transcriptionally most similar co‐express these genes, so donor/receiver sets overlap strongly in this tight neighborhood.^[^ ^52^ ^]^ On the *k* = 12 graph we observe GAL‐TH, corresponding to the galaninergic modulation of dopaminergic neurons which is an example of cross‐subtype interactions between biologically coupled but physically separated clusters.^[^ ^53^ ^]^ Finally, on the *k* = 15 graph we revealed long‐range neuromodulatory programs such as PNOC–OPRL1 and SLC18A2–HTR2C, which arise from distributed nociceptin and monoaminergic signalling networks.^[^ ^54^ ^]^

### 2.6 Evaluating MCIST on StereoSeq, STARmap, and seqFISH Data

StereoSeq is a high‐resolution spatial method with the ability to achieve sub‐cellular resolution by combining with microscopy while sequencing thousands of genes.^[^ ^55^ ^]^ Specifically, Table S1 (Supporting Information) shows that some StereoSeq datasets have a very large number of samples coupled with very high dimensions, which can be a challenge to many methods, such as SpaceFlow. Additionally, STARmap is an experimental technology capable of generating three‐dimensional images of gene expression, offering a more comprehensive view of tissue architecture. The STARmap data used in this study contains only one dataset with 1020 genes and 1207 samples.^[^ ^56^ ^]^

**Figure 6:**
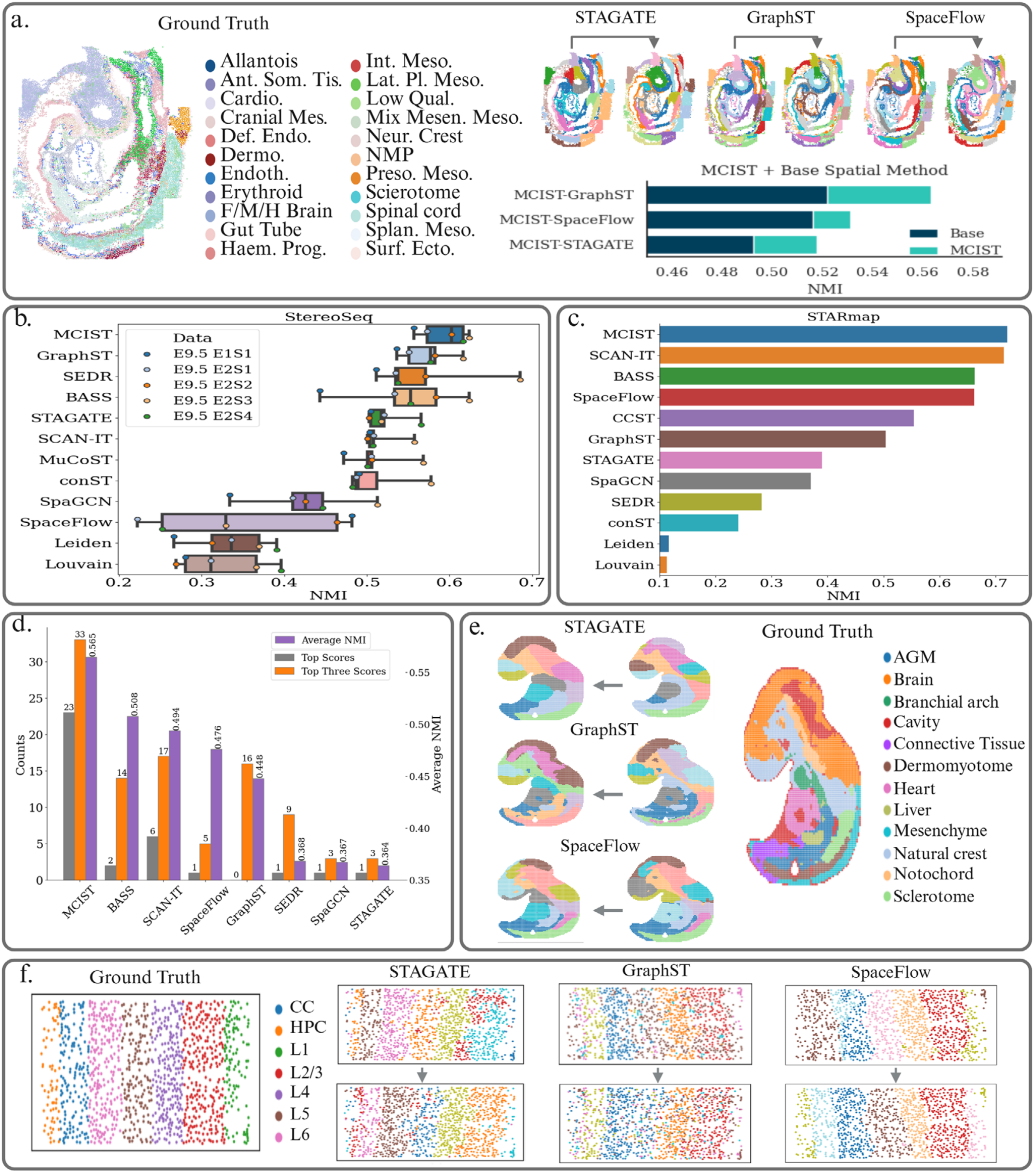
a) Performance of MCIST variants on single cell resolution seqFISH data demonstrates the improvement in performance across deep learning modules for newer generation sequencing technology. b) Performance comparison on the StereoSeq data over seven datasets. MCIST, when utilizing the GraphST deep learning model, was able to achieve state‐of‐the‐art results. Results of other leading methods were adopted from the latest benchmarking study.^[^ ^21^ ^]^ Methods are ranked according to average performance. Results for samples E10.5 E1S3 and E10.5 E2S1 are available in Table S12 (Supporting Information) for fairness as they were not tested on all methods in the latest clustering benchmarking study.^[^ ^21^ ^]^ c) Comparisons of NMI scores on the STARmap data. MCIST was paired with the SpaceFlow deep learning model and was able to outperform the next best method by roughly 1%. Results of other methods were adopted from the latest benchmarking study.^[^ ^21^ ^]^ Methods are ranked according to average performance. d) Average results over all 37 datasets. The number above a specific bar indicates the number that the method was ranked the best (grey), or top three best (orange), among all methods in 37 datasets. For example, MCIST was ranked number one in 23 out of 37 datasets and was a top three method on 33 datasets. The average NMI scores over all 37 datasets are also available in purple, with MCIST achieving the highest average score by a margin of over 11%. e) Comparisons of MCIST performance on StereoSeq data when combining with different deep learning methods using the same set of parameters. MCIST was able to improve the performance of STAGATE and GraphST but did not significantly impact the performance of SpaceFlow. f) Comparisons of MCIST performance on STARmap when combining with different deep learning methods using the same set of parameters. MCIST was able to considerably improve the performance of STAGATE and SpaceFlow but did not significantly impact the performance of GraphST.

In **Figure** **6**b, we highlight the results of 11 different methods in the StereoSeq data. We observe similar improvements in performance from MCIST in this case. MCIST was able to achieve a mean NMI score of 0.572 in seven datasets when paired with GraphST, an improvement of 5.8% over the reported results for the baseline deep‐learning method. We note that SpaceFlow did not perform well on the StereoSeq data.

In Table S12 (Supporting Information), we include the results for additional two datasets that were tested on only a partial subset of the methods in the latest benchmarking study,^[^ ^21^ ^]^ and find that MCIST is able to achieve good performance on these datasets as well. When combined with STAGATE, the average performance was an NMI score of 0.568, which is an average improvement of 12.4% over the base spatial model over the first seven datasets. However, we achieved superior results by combining with GraphST, where there was an average improvement of 5.8% over the base method, resulting in state‐of‐the‐art performance. A complete account of the results for MCIST and other methods on the StereoSeq platform can be found in Table S11 (Supporting Information).

In Figure 6a, we include an analysis on the next generation single cell resolution seqFISH dataset of a whole‐mount sagittal cryosection of a mid‐gestation mouse embryo.^[^ ^57^ ^]^ The embryo‐stage seqFISH data set is partitioned into 22 spatial domains. Listed alphabetically, they are Allantois (Allantois), Anterior somitic tissues (Ant. Som. Tis.), Cardiomyocytes (Cardio.), Cranial mesoderm (Cran. Mes.), Definitive endoderm (Def. Endo.), Dermomyotome (Dermo.), Endothelium (Endoth.), Erythroid (Eryth.), Forebrain / Midbrain / Hindbrain (FM/H Brain), Gut tube (Gut Tube), Haemato‐endothelial progenitors (Haem. Prog.), Intermediate mesoderm (Int. Meso.), Lateral plate mesoderm (Lat. Pl. Meso.), Low‐quality spots (Low Qual.), Mixed mesenchymal mesoderm (Mix Mes. Meso.), Neural crest (Neur. Crest), Neuromesodermal progenitors (NMP), Presomitic mesoderm (Presom. Meso.), Sclerotome (Sclerotome), Spinal cord (Spinal Cord), Splanchnic mesoderm (Splan. Meso.), and Surface ectoderm (Surf. Ecto.). Across STAGATE, GraphST, and SpaceFlow, the inclusion of transcription similarity graph structure via MCIST improved the performance of spatial domain clustering by 5.1%, 7.9%, and 2.9% respectively according to NMI. Specifically, for each base deep learning model, the corresponding MCIST clusters were able to more accurately segment the Forebrain/Midbrain/Hindbrain region along with the Cranial mesoderm which hugs its lower edge. This region makes up the pharyngeal‐arch mesenchyme and craniofacial muscles, which gives rise to the entire nervous system anterior to the spinal chord.^[^ ^58^ ^]^ Additionally, we observe that MCIST when paired with GraphST enables the clustering to identify the Erythroid region, a crescent beneath the outer boundary on the right of the tissue, which was missed by the base deep learning model. However, we note that with the large size of this sample (roughly 19 thousand cells), MCIST did encounter significant runtime increases due to the repeated eigenvector calculations and matrix multiplications in the Topological PCA algorithm. On an AMD EPYC 7H12 “Milan” CPU with 64 physical cores (no SMT) with 256GiB of allocated memory, the MCIST code completed in roughly 3 h, compared to an average of just 20 minutes for the base deep learning algorithms. We conclude that while MCIST does offer performance improvements and scales very easily on smaller Visium/MERFISH/etc. data; on very large data, such as seqFISH and StereoSeq, additional computational advancements including further parallelization or re‐implementation in C++ to allow fine‐grained control over memory allocation would be extremely beneficial.

Finally, we present a comparison of the performance of 12 computational methods on the STARmap data in Figure 6c. MCIST was able to slightly edge out the second best deep learning technique, SCAN‐IT, by a margin of almost 1% when combined with SpaceFlow deep learning. Compared to SpaceFlow itself, MCIST improves performance by a considerable margin of 8.9%, further illustrating the importance of cell–cell interaction analysis as well as the generalization capacity of MCIST to utilize multiple different deep learning approaches. As expected, the two non‐spatial methods, Louvain and Leiden, did not perform well. A complete account of the results for MCIST and other methods on the STARmap platform can be found in Table S14.

## 3 Discussion

### 3.1 Overall Performance

Recent years have witnessed a surge in new methods for spatial transcriptomic data analysis, due to the growing importance of spatial transcriptomic experiments in biological science. However, there is no analysis method that accounts for multiscale cell–cell interactions, which are omnipresent in biological systems. As a result, none of the current spatial transcriptomic analysis methods is able to effectively deal with all spatial transcriptomic data acquired from different experimental platforms.^[^ ^21^ ^]^ In this work, we propose MCIST, the first method to address multiscale cell‐cell interactions in spatial transcriptomic analysis. We have amassed a total of 37 datasets involving a wide variety of spatial transcriptomic technologies to assess the performance of MCIST against a large number of other state‐of‐the‐art spatial methods, both deep learning and probability‐based. Due to the diverse nature of spatial transcriptomic technologies, none of the existing methods is able to achieve satisfactory performance on all datasets. The specific results for various datasets are presented in Supporting Information Tables S8‐S14. Overall, when MCIST utilized GraphST for the high dimensional Visium and StereoSeq platforms, and SpaceFlow for the relatively low dimensional ST, MERFISH, BaristaSeq, and STARmap platforms, it achieved the best performance on 23 out of 37 datasets‐ 17 more than the next method, SCAN‐IT, in terms of dataset counts, and 21 more than the second‐best performing method, BASS, in terms of average NMI, as shown in Figure 6d. MCIST was also one of the top three performing methods on 33 datasets, 16 more than the next method, SCAN‐IT. In general, each method among BASS, SCAN‐IT, GraphST, SEDR, SpaGCN, SpaceFlow, and STAGATE attain the best score for at least one dataset, indicating their merits in spatial transcriptomics data analysis. We point out that several methods, such as conST and CCST, which were able to achieve top or top three performance on some of the datasets are excluded from the figure because they were not validated on all 37 datasets in the recent benchmarking study. Leiden and Louvain, meanwhile, are baseline methods and therefore were also excluded from the comparison. Some other methods that showed up in the earlier analysis are not included in the overall comparison because they are not applicable to all of the 37 datasets, such as BayesSpace, which is only compatible with spot resolution data. We find that MCIST is able to achieve a mean performance of 0.565 for NMI in all datasets, outperforming the second‐best BASS by 11.2%. We note that this outstanding performance accounts for different choices of deep learning and clustering algorithms across different spatial platforms. In the Supporting Information Tables, we provide the results for MCIST using SpaceFlow, STAGATE, and GraphST each on all 37 datasets. When combined with STAGATE, the most scalable algorithm, and Mclust clustering for all 37 datasets, MCIST is still able to achieve state‐of‐the‐art results, outperforming BASS by more than 4%. MCIST is also an outstanding method in revealing biological processes in pseudotime dynamics (Figure 2b) and signaling pathways with differentially expressed genes (Figure 4c,d).

### 3.2 Alternative MCIST Implementations

We emphasize in Figure 6e,f that multiscale cell‐cell interactive topological embeddings can be aligned with those from multiple spatially resolved deep learning techniques. The ability to generalize MCIST means that we can benefit from the strengths of different deep learning approaches and thus enhance their performance. GraphST, for example, was able to significantly outperform STAGATE and SpaceFlow on the high dimensional data such as StereoSeq and Visium. In the latest benchmarking study for the StereoSeq sample, GraphST showed high concordance with known marker genes of the major organs and identified two clusters for the embryo heart's atrium and ventricle chambers.^[^ ^21^ ^]^ STAGATE, meanwhile, was unable to identify the two sections of the heart when the number of clusters matched the ground truth, and could not reliably identify the other organs either. We therefore found it more beneficial to pair MCIST with GraphST on this data to benefit from its superior performance. GraphST benefits here from the use of graph self‐supervised contrastive learning and decoding to strengthen the latent representation.^[^ ^15^ ^]^ In conjunction with the manifold structure informed features produced by topological PCA, we found that GraphST becomes a very powerful tool for identifying accurate clusters in high‐resolution or high dimensional spatial transcriptomics data. We note, however, that the topological features did not significantly impact the performance of GraphST on the STARmap data in Figure 6f. Other deep learning methods, meanwhile, demonstrated considerable improvements in performance. MCIST paired with the SpaceFlow, for example, improved performance by 8.9% on this data. We generally found that SpaceFlow performed better in MCIST for the lower dimensional data, such as ST, MERFISH, BaristaSeq, and STARmap. SpaceFlow utilizes a deep graph infomax framework, a contrastive learning strategy, to enhance the ability of GCNs to capture the cell neighborhood microenvironment.^[^ ^10^ ^]^ By randomly permuting the expression profiles of spots in the spatial graph and utilizing a discriminator SpaceFlow forces the embedding layer to learn global patterns and reject random spatial expression patterns. We found MCIST achieved significantly superior results pairing with this module compared to a GATE on the STARmap data. As we have mentioned, overall MCIST achieved superior results on 23/37 datasets, although it would be insightful to note the properties of the methods which were able to consistently outperform MCIST on certain data. Specifically, SCAN‐IT was able to outperform MCIST on 6 datasets as shown in the Supporting Information Tables S8– S14. Specifically, SCAN‐IT achieved superior performance primarily on the MERFISH and BaristaSeq platforms which are characterized by low gene targeting depth. The inclusion of the histology multi‐view perspective was likely especially useful in this case as a means of introducing new information when relatively little is available from gene expression values alone due to low gene targeting depth. Additionally, the recent PearlST model was found to achieve excellent results in a variety of spatial domain detection and pseudotime inference tasks by uniting contrastively learned histological features with a PDE based diffusion model to learn multi‐view informed latent embeddings. They also are able to infer downstream functions by associating up‐regulated LR pairs to the latent features, indicating a list of target genes for downstream analysis.^[^ ^16^ ^]^ SCAN‐IT reformulates the spatial domain detection problem as an image segmentation task, where cells mimic pixels and expression values mimic the RGB channel.^[^ ^8^ ^]^ SCAN‐IT also utilizes a deep graph infomax framework to train a GCN to produce multiple embeddings which are used to derive a consensus representation. Specifically, the use of a consensus distance matrix is very similar in nature to the construction of the accumulated spectral graph from topological PCA. This further emphasizes the fact that, whether in trying to accommodate the randomness of deep learning modules, or in trying to gain a more thorough spatial view of the cell‐interaction structure in the data, multiscale analysis holds considerable potential for further driving improvements in the field. A natural development in the field to this end would be the development of a coherent deep learning method for MCIST, either via the development of a novel spatially resolved encoder or the implementation of similar graph neural network methods for the cell similarity relations.

### 3.3 Ensemble Clustering and Optimization

Multiscale topological PCA involves several hyperparemters, specifically the connectivity weightings in the *L*_P_ that balance different scales, along with two other parameters. The full parameter optimization would be time consuming. We thus take an ensemble of multiple different scales of connectivity combinations, rather than searching for the optimal parametrization as described in Supporting Information Section S3. Additionally, we recognize that in spatial transcriptomics data there are generally no available ground truth annotations to guide any parameter selection. For example, our 38th dataset included in the Supporting Information, a Visium Breast Tumor dataset, lacks pathologist annotated labels. This motivates the use of a metric that does not depend on ground truth labels but correlates well with accuracy in classification problems and clustering convergence. We previously proposed the Residue‐Similarity Index (RSI) for evaluating clustering methods.^[^ ^33^ ^]^ Tests on various datasets utilized in this study demonstrate a statistically significant Pearson correlation coefficient of 0.6 between RSI and ARI and 0.56 between RSI and NMI across several of the DLPFC datasets. On the MERFISH data, we observe a statistically significant Pearson correlation coefficient of 0.43 between RSI and ARI and 0.53 between RSI and NMI. We therefore utilized this metric to develop an unsupervised learning optimization approach for MCIST. More information regarding this process and a performance comparison of various methods on the Visium Breast Tumor dataset can be found in Tables S15– S17, Figures S3 and S4, and Section S7 and S12 (Supporting Information). For robustness, it is generally beneficial to consider a distribution of parameter values, and take a consensus among the different induced representations. We therefore produce multiple tPCA embeddings, each emphasizing different combinations of filtration scales, and clustered each separately to then perform Agglomerative clustering to delineate the final spatial domains. We then also have the choice of which clustering algorithm to use in the Agglomerative clustering‐ notably, either the Mclust or Leiden algorithms. In this study, we utilized the Leiden algorithm for the BaristaSeq, StereoSeq, and MERFISH data, and the Mclust algorithm for the Visium, ST, and STARmap data. In the case of the STARmap data, spatial domain detection was performed using only the embedding which maximized the RSI score to showcase the benefit of optimizing performance. As stated before, in the Supporting Information we present the collected results over all 37 datasets for each possible deep learning and clustering algorithm combination in MCIST.

### 3.4 MCIST is Adaptive to Diverse Spatial Platforms

We have observed from the collection of 37 benchmark datasets in 6 different spatial technologies that MCIST is not only compatible with a wide variety of spatial transcriptomics data but also capable of achieving the best quantitative results. MERFISH, STARmap, and StereoSeq are each notable for their high single‐cell resolution. Single‐cell resolution enables the distinction between different cell types and states within a complex tissue environment, aiding in a better understanding of how cells interact with their neighbors. StereoSeq can even achieve subcellular resolution, allowing researchers to pinpoint the location of RNA molecules within different parts of a single cell, providing insight into important cellular processes such as RNA transport. Spatial technologies will continue to improve their spatial resolution, enlarge their data size, and reach higher gene dimensionality in the near future, which poses a challenge to current spatial transcriptomic methods. MCIST is designed to tackle multiscale spatial heterogeneity and high intrinsic gene dimensions, offering a promising method for future spatial transcriptomic analysis.

## 4 Experimental Section

### 4.1 Data Preprocessing and Statistics

The raw count expression matrix of our ST data was preprocessed via the following standard pipeline. First, genes with detected expression in fewer than 3 cells and cells with expression of fewer than 100 genes are discarded. Subsequently, normalization was performed, where the expression of each gene is divided by total expression in that cell, so that every cell has the same total count after normalization. Then, the normalized expression was multiplied by a given scale factor (1e4 by default) and log‐transformed. The log‐transformed expression matrix of the top 3000 highly variable genes (HVGs) was then selected as the input for subsequent analysis. A statistical summary of all data utilized in this study can be found in the Supporting Information (S1). For differential gene expression analysis, the Scanpy library was used to perform a Wilcoxon rank‐sum test with a Benjamini–Hochberg *p* value correction. We then considered the co‐expressed DEGs after filtering for an adjusted *p* value for domain‐specific marker genes of 0.05.

### 4.2 Multiscale Topological PCA—Manifold Regularization

For a given gene expression matrix $X\in R^{M\times N}$ with *N* spots and *M* genes, one wishes to express $\mathbf{*X}$* in a lower‐dimensional form, say in *m* dimensions, via matrices $U\in R^{M\times m}$ and $Q\in R^{N\times m}$, where matrices $\mathbf{U}$ and *$\mathbf{Q}$* denote the principal components and projected data matrix respectively. Sparse PCA^[^ ^59^ ^]^ encourages spots with similar expression profiles to share a common representation via the minimization of the L_2, 1_ norm of the projected data matrix.^[^ ^60^ ^]^ This has the effect of eliminating the contribution of genes which do not contribute significantly to the variance within a cluster of spots. Therefore, Sparse PCA can significantly reduce signal noise in the dimensionality reduction. It is formulated as:

(1) $$\begin{array}{c} \min_{U,Q}∥X-UQ^{T}{∥}_{2,1}+\beta {∥Q∥}_{2,1},\;\text{s.t.}\;U^{T}U=I_{m} \end{array}$$

Laplacian embedding, meanwhile, utilizes neighbor graphs so that similar samples in the higher‐dimensional space are closer in the lower‐dimensional embedding.^[^ ^61^ ^]^ Let *G*(*V*, *E*, *W*) be a nearest neighbor graph, where *V* is the set of vertices, *E* is the edge, and ω are the weights of the edges. *E* can be defined as $E=\{\left(x_{j},x_{i}\right):x_{i}\in N_{k}\left(x_{j}\right)\;\text{or}\;x_{j}\in N_{k}\left(x_{i}\right)\}$, where $N_{k}\left(x_{j}\right)$ is the *k*‐nearest neighbors of sample *j* under some metric. For pairs of points in the edge set, one can define a weight satisfying the following two properties,

(2) $$\begin{array}{cc} & \Phi \left(x_{i},x_{j}\right)\to 1\;\text{as}{∥x_{i}-x_{j}∥}_{2}\to 0 \end{array}$$

(3) $$\begin{array}{cc} & \Phi \left(x_{i},x_{j}\right)\to 0\;\text{as}{∥x_{i}-x_{j}∥}_{2}\to \infty , \end{array}$$

where ‖**x**_*i*_ − **x**_*j*_‖_2_ is the *L*_2_ distance between cell *i* and cell *j* in the gene expression space. These properties are satisfied by a class of radial basis functions. In this work, we utilize the Gaussian kernel as the edge weights,

(4) $$W_{ij}=\left\{\begin{array}{cc} e^{-∥x_{i},x_{j}{∥}_{2}^{2}/\eta } & \;\text{if}\;x_{j}\in N_{k}\left(x_{i}\right)\\ 0, & \;\text{otherwise}. \end{array}\right.$$

The matrix *W* is known as the weighted adjacency matrix. Here, η∈R defines the geodesic distance, or the width of the Gaussian kernel. We can then define the graph Laplacian *L*, by taking

(5) $$\begin{array}{c} L=D-W, \end{array}$$

(6) $$\begin{array}{c} D_{ii}=\sum_{j=1}^{N}W_{i,j}, \end{array}$$

where *D* is the degree matrix, defined as the row sum of *W*, which shows the total connectivity of the vertex *i*. Laplacian graph provides a graphical embedding, which can be used as a regularization for PCA.^[^ ^60^ ^]^

In order to incorporate manifold regularization into the PCA framework, consider the distance ‖**q**_*i*_ − **q**_*j*_‖^2^, where **q**_*i*_ and **q**_*j*_ correspond to the lower‐dimensional representation of samples **x**_*i*_ and **x**_*j*_, respectively. Using the graph weights *W*_*ij*_, it is desirable that if *W*_*ij*_ → 1, i.e., **x**_*i*_ and **x**_*j*_ are similar, then ‖**q**_*i*_ − **q**_*j*_‖ → 0. Alternatively, if *W*_*ij*_ → 0, i.e., **x**_*i*_ and **x**_*j*_ are dissimilar, ‖**q**_*i*_ − **q**_*j*_‖^2^ → ∞. Using this guide, the following is meant to be minimized.

(7) $$\begin{array}{cc} R & =\frac{1}{2}\sum_{ij}^{N}W_{ij}{∥q_{i}-q_{j}∥}^{2} \end{array}$$

(8) $$\begin{array}{cc} & =\frac{1}{2}\left(\sum_{ij}^{N}W_{ij}\left(q_{i}^{T}q_{i}+q_{j}^{T}q_{j}\right)\right)-\sum_{ij}^{N}W_{ij}q_{i}^{T}q_{j}^{T} \end{array}$$

(9) $$\begin{array}{cc} & =\sum_{i}^{N}D_{ii}q_{i}^{T}q_{i}-\sum_{ij}^{N}W_{ij}q_{i}^{T}q_{j}^{T} \end{array}$$

(10) $$\begin{array}{cc} & =\text{Tr}\left(Q^{T}DQ\right)-\text{Tr}\left(Q^{T}WQ\right) \end{array}$$

(11) $$\begin{array}{cc} & =\text{Tr}(Q^{T}LQ). \end{array}$$

It was noted that the optimal *$\mathbf{Q}$* here which minimizes this objective function is the matrix of eigenvectors of the symmetric graph Laplacian, and is therefore an orthogonal matrix. To combine this objective function with that of sparse PCA would thus necessitate the imposition of an orthogonality constraint on *$\mathbf{Q}$* rather than *$\mathbf{U}$*.

### 4.3 Multiscale Topological PCA—Persistent Lapacians and Multiscale Topological PCA

The above framework, however, still lacks the ability to recognize the stability of topological features at multiple scales.^[^ ^62^ ^]^ To this end, we turn to persistent Laplacian regularization. Like persistent homology, persistent spectral graph theory tracks the birth and death of topological features of data as they change over scales. This analysis can be performed via a filtration procedure on our data to construct a family of geometric structures.^[^ ^62^ ^]^ Then, one can extract the topological properties of each configuration by its corresponding Laplacian matrix.

First, we briefly review the notion of a simplex, simplicial complex, *q*‐chain, and boundary. A 0‐simplex is a vertex, a 1‐simplex is an edge, a 2‐simplex is a triangle, and so on. Generally, a *q*‐simplex can be considered, which we denote σ_*q*_. A simplicial complex is then a means of approximating a topological space by gluing together the faces of these simplices. More formally, a simplicial complex *K* is a collection of simplices such that:

1. If σ_*q*_ ∈ *K* and σ_*p*_ is a face of σ_*q*_ then σ_*p*_ ∈ *K*.
2. The nonempty intersection of any two simplices is a face of both simplices.

A graph therefore can be viewed as the skeleton of a simplicial complex, made up of only 0‐simplexes and 1‐simplexes. In general, a *q*‐chain is defined as a formal sum of *q*‐simplices in a simplicial complex *K* with coefficients in $Z_{2}$. The set of *q*‐chains has a basis in the set of *q*‐simplices in *K*, and this set forms a finitely generated free Abelian group *C*_*q*_(*K*). The boundary operator was defined as a homomorphism relating the chain groups, ∂_*q*_: *C*_*q*_(*K*) → *C*_*q* − 1_(*K*). The boundary operator is defined as:

(12) $$\partial_{q}\sigma_{q}=\sum_{i=0}^{q}{\left(-1\right)}^{i}\sigma_{q-1}.$$

where σ_*q* − 1_ is a *q* − 1 simplex induced by the removal of the $i$th vertex from the original simplex. The sequence of chain groups connected by this homomorphism is then a chain complex:

(13) $$\text{…}\overset{\partial_{q+1}}{\to }C_{q}\left(K\right)\overset{\partial_{q}}{\to }C_{q-1}\left(K\right)\overset{\partial_{q-1}}{\to }\text{…}$$

It is well known that the boundary operator and the chain complex associated with a simplicial complex give the number of *q*‐dimensional holes in that topological space. Specifically, the *q*th homology group is defined as *H*_*q*_ = ker∂_*q*_/Im∂_*q*_. This is also known as the *q*th Betti number, β_*q*_. The matrix representation of the *q*th boundary operator with respect to the standard basis in *C*_*q*_(*K*) and *C*_*q* − 1_(*K*) is given as $B_{q}$. In addition, considering the homology of the topological space, its cohomology can also be considered. To that end, the adjoint operator of ∂_*q*_ is defined as:

(14) $$\partial_{q}^{*}:C_{q-1}\left(K\right)\to C_{q}\left(K\right),$$

and the transpose of $B_{q}$, denoted $B_{q}^{T}$, is the matrix representation of $\partial_{q}^{*}$ with respect to the same basis. Now, the *q*‐combinatorial Laplacian matrix is defined as:

(15) $$L_{q}:=B_{q+1}B_{q+1}^{T}+B_{q}^{T}B_{q}.$$

Specifically, the structure could be considered of the skeleton graph, or the cell–cell interaction graph. Equivalent to the graph Laplacian is the *L*_0_ combinatorial Laplacian, defined as:

(16) $$L_{0}=B_{1}B_{1}^{T}=D-A$$

The harmonic spectrum of the *q*‐combinatorial Laplacian matrix reveals the dimension of the *q*th homology group, or the number of *q*‐dimensional holes in the simplicial complex. The non‐harmonic spectrum then reveals further homotopic shape information.^[^ ^62^ ^]^ Intuitively, β_0_ reveals the number of connected components in *K*, β_1_ reveals the number of loops in *K*, and β_2_ reveals the number of 2D voids in *K*.

However, this framework is confined to the analysis of only a single simplicial complex or graph, and the connectivities at only a single scale. To enrich our spectral information, Persistent spectral graph theory proposes creating a sequence of geometric configurations by varying a filtration parameter:^[^ ^62^ ^]^

(17) $$\left\{\emptyset \right\}=K_{0}\subseteq K_{1}\subseteq \text{…}\subseteq K_{p}=K$$

For each subcomplex/subgraph *K*_*t*_ we can denote its chain group to be *C*_*q*_(*K*_*t*_), and the *q*‐boundary operator $\partial_{q}^{t}:C_{q}\left(K_{t}\right)\to C_{q-1}\left(K_{t}\right)$. By convention, we define *C*_*q*_(*K*_*t*_) = {0} for *q* < 0 and the *q*‐boundary operator to then be the zero map. The boundary operator and adjoint boundary operator are otherwise defined similarly as before for each *K*_*t*_ in the sequence, which allows us to define chain complexes.

Next, persistence was introduced to the Laplacian spectra. Define the subset of $C_{q}^{t+p}$ whose boundary is in $C_{q-1}^{t}$ as $C_{q}^{t,p}$, assuming the natural inclusion map from $C_{q-1}^{t}\to C_{q-1}^{t+p}$.

(18) $$C_{q}^{t,p}:=\left\{\beta \in C_{q}^{t+p}|\partial_{q}^{t,p}\left(\beta \right)\in C_{q-1}^{t}\right\}.$$

On this subset, one may define the *p*‐persistent *q*‐boundary operator denoted by ${\hat{\partial }}_{q}^{t,p}:C_{q}^{t,p}\to C_{q-1}^{t}$ and corresponding adjoint operator ${\left({\hat{\partial }}_{q}^{t,p}\right)}^{*}:C_{q-1}^{t}\to C_{q}^{t,p}$, as before. The matrix representation of the *p*‐persistent *q*‐boundary operator in simplicial basis is then $B_{q+1}^{t,p}$, and the matrix representation of the adjoint operator is again the transpose. This allows to define the *q*‐order *p*‐persistent Laplacian matrix as:

(19) $$L_{q}^{t,p}:=B_{q+1}^{t,p}{\left(B_{q+1}^{t,p}\right)}^{T}+{\left(B_{q}^{t}\right)}^{T}B_{q}^{t}.$$

The multiplicity of zero in the spectrum of $L_{q}^{t,p}$ might be utilised as the *q*'th order *p*‐persistent Betti number $\beta_{q}^{t,p}$ which counts the number of (independent) *q*‐dimensional voids in *K*_*t*_ that still exists in *K*_*t* + *p*_.^[^ ^62^ ^]^ Then one could see how the *q*'th‐order Laplacian is actually just a special case of the *q*'th‐order 0‐persistent Laplacian at a simplicial complex *K*_*t*_, or rather, at a snapshot of the filtration. The multiscale topological structure of a sequence of cell‐cell interaction graphs can then be described by a collection of these snapshots:

(20) $$\left\{L_{0}^{0,0},L_{0}^{1,0},L_{0}^{2,0},\text{…},L_{0}^{p,0}\right\}$$

Vietoris‐Rips complexes were calculated by varying a filtration parameter on the weighted entries of our Laplacian matrix, which correspond to the weighted edges in this graph structure. By gradually increasing a distance threshold, a sequence of subgraphs was induced to analyze. In previous work on persistent Laplacian‐enhanced PCA, a convenient computational method was provided for this. For a graph Laplacian matrix *L*, observe:

(21) $$L=\left(l_{ij}\right),l_{ij}=\left\{\begin{array}{c} l_{ij},i\neq j,i,j=1,\text{…},n\\ l_{ii}=-\sum_{j=1}^{n}l_{ij}. \end{array}\right.$$

We then may consider a kNN‐induced filtration, where we induce a sequence of subgraphs by varying the number of nearest neighbors in a cell–cell interaction kNN graph. The kNN induced filtration is particularly attractive for gene expression data given that its high sparsity and highly nonlinear relations can significantly affect distance measurements, leading to difficulties in establishing meaningful connections based solely on set distance thresholds. A kNN based filtration overcomes these difficulties by ensuring that connections are assigned or removed consistently at each scale. Therefore, set the *k* ^*th*^ Persistent Laplacian *L* ^*k*^, *k* = 1, …, *p*:

(22) $$L^{k}=\left(l_{ij}^{k}\right),\;l_{ij}^{k}=\left\{\begin{array}{c} -1,\;\text{if}\;i\neq j\;\text{and}\;x_{j}\in N_{k}\left(x_{i}\right)\\ 0,\;\text{otherwise} \end{array}\right.$$

Here,

(23) $$l_{ii}^{k}=-\sum_{j=1}^{n}l_{ij}^{k}.$$

We then weight each *L* ^*k*^ in the sequence and sum to consolidate each subgraph into a single term, denoted *L*_P_, which can be thought of as an accumulated spectral graph. This new term should encode the persistence of topological features as the filtration progresses over multiple scales,

(24) $$L_{P}:=\sum_{k=1}^{p}\zeta_{k}L^{k}.$$

The optimal ζ weightings are hyper parameters which may be obtained via a grid search. Ideally, which scales of connectivity contribute the most important information to this analysis should be recognized, and place greater emphasis on that corresponding Laplacian matrix in the sum. In practice, it is sufficient to use ζ values of either 0 or 1, which can be thought of as “turning on or off” the connectivity at that scale. Given this enhanced multiscale view of the data, the *L*_P_ term was substituted for the graph Laplacian in the Laplacian embedding formula and combine with Sparse PCA to formulate what we have previously called “topological PCA,” defined by:

(25) $$\begin{array}{c} \min_{U,Q}∥X-UQ^{T}{∥}_{2,1}+\beta {∥Q∥}_{2,1}+\gamma \text{Tr}\left(Q^{T}\left(L_{P}\right)Q\right),\;\text{s.t.}\;Q^{T}Q=I_{m} \end{array}$$

### 4.4 Spatially Informed Deep Learning

Recently, a variety of deep learning techniques have been developed for accounting spatial information in gene expression analysis. In this section, some of these notable methods are briefly described, which were employed in MCIST throughout this study.

### 4.5 Spatially Informed Deep Learning—STAGATE

For convenience, the STAGATE deep learning architecture might be utilized in MCIST due to its efficiency, as done in the downstream analysis of the ST breast tumor dataset H1. STAGATE uses a graph attention auto‐encoder framework to identify spatial domains by integrating spatial information and gene expression profiles. The spatial information is used to construct a spatial network, from which the neighbors of a spot can influence its latent representation via an attention mechanism.^[^ ^9^ ^]^

### 4.6 Spatially Informed Deep Learning—SpaceFlow

For benchmarking the low dimensional MERFISH, BaristaSeq, ST, and STARmap data, we utilized the SpaceFlow deep learning architecture in MCIST. SpaceFlow generates spatially‐consistent low‐dimensional embeddings through expression similarity and spatial information via spatially regularized deep graph networks with an added discriminator layer to discern global expression patterns.^[^ ^10^ ^]^

### 4.7 Spatially Informed Deep Learning—GraphST

For benchmarking the high dimensional Visium and StereoSeq data, the GraphST deep learning architecture was utilized in MCIST. GraphST is a graph self‐supervised contrastive learning method that combines graph neural networks with augmentation‐based self‐supervised contrastive learning to learn representations of spots for spatial clustering by encoding both gene expression and spatial proximity.^[^ ^15^ ^]^

### 4.8 MCIST

MCIST generates embeddings obtained from multiscale topological PCA to encode the intrinsic manifold structure of cell–cell interactions in the gene expression space and to provide a multiscale description of these interactions as characterized by expression vector correlations. These embeddings are further paired with a variety of deep‐learning based spatial techniques such as STAGATE, SpaceFlow, or GraphST to achieve the state‐of‐the‐art performance for spatial domain detection. By aligning the embeddings of these methods, MCIST can extend their complementary advantages.

To extract the most benefit from tPCA, we fix several of the model parameters in Equation (25) to simultaneously preserve the inherent graph relations along with the data sparsity. Specifically, the geometrical structure capture term γ and the sparsity enforcement term β are both set to 1. In previous work we have demonstrated that tPCA performance is stable for hyper‐parameter values ranging from 1*e* − 5 to 1*e*5. We then considered values of 0 or 1 for each of the connectivity weightings, which can be thought of as “turning on or off” that scale of interactions, providing feature sets reflecting different combinations of kNN graph scales for ensemble learning. With four filtration steps on our kNN cell‐cell interaction graph, corresponding to *k* = 15, 12, 9, 6 neighbors, a consensus was taken with different combinations of each scale. Each respective scale carries with its own biological motivation. The broadest scale, *k* = 15, is the default number of neighbors in the Scanpy pipeline as well as for the popular UMAP method, given that community‐level structures or broad cell classes generally sit in this range.^[^ ^4^, ^63^ ^]^ At the end, *k* = 6, we narrowly focus on immediate transcriptional neighbors, or cells that share the most similar expression profiles and are likely in very similar states. By stepping through *k* = 15, 12, 9, 6 we sample evenly (and roughly evenly in log‐space) from large‐scale to small‐scale structure, avoiding an over‐concentration of analysis at either extreme and ensuring a smooth transition between broad cell classes and local cellular niches. After embedding the data with the various connectivity combinations, denoted *k*_1_, …, *k*_*p*_, the feature set of topological PCA features $\left[Q_{\text{Co-Expression}}^{\left(k_{1}\right)},\text{…},Q_{\text{Co-Expression}}^{\left(k_{p}\right)}\right]$ was obtained. MCIST will then align these features with the output of a spatially resolved deep learning encoder, denoted *Q*_Spatial_, to obtain the new feature set which maximally extracts the shared biological signal from the physical and transcription adjacencies, aligning with our intuition of cell interactions manifesting as some combination of spatial relation along with transcription correlation. This feature set is then given as $\left[Q_{1},\text{…},Q_{p}\right]$ where each $Q_{i}$ is produced via a Canonical Correlation Analysis to align the multi‐view spatial‐transcription relation embeddings. Specifically, an effort is made to build a solution which solves,

(26) $$\begin{array}{c} \left({\left(Q_{\text{Spatial}}\right)}^{T}Q_{\text{Co-Expression}}^{\left(k_{i}\right)}\right)\left({\left(Q_{\text{Co-Expression}}^{\left(k_{i}\right)}\right)}^{T}Q_{\text{Spatial}}\right)q=\lambda q \end{array}$$

where λ is the squared correlation between *q* ^*T*^ *Q*_Spatial_ and $p^{T}Q_{\text{Co-Expression}}^{\left(k_{i}\right)}$ and *p* is given by

(27) $$\begin{array}{c} \left({\left(Q_{\text{Co-Expression}}^{\left(k_{i}\right)}\right)}^{T}Q_{\text{Spatial}}\right)q/\sqrt{\lambda } \end{array}$$

These *q*, *p* are the first pair of canonical variables capturing the majority of shared correlation or biological signal between the deep learning and tPCA representations. This solution was thus repeated to obtain a set of uncorrelated vectors each capturing the shared correlation structure in the descending order, which then combine to form our feature matrix $Q_{k_{i}}$ for the *i* ^th^ tPCA subgraph.

We then performed unsupervised clusterings on each $Q_{k_{i}}\in \left[Q_{k_{1}},\text{…},Q_{k_{p}}\right]$ using either the Mclust or Leiden algorithm to obtain a set of clustering results for each cell. Each clustering result is then used to construct a co‐association matrix, which counts how often each pair of samples is assigned to the same cluster across all parameter configurations. The co‐association matrix is then used to infer pairwise cell distances which can be used for Agglomerative clustering/ensemble learning to give us our final reconstructed spatial domains.

In cases where we wish to use a single embedded representation of the gene expression data for downstream analysis, such as in Section 2.4, the connectivity weightings could be partially optimized by measuring which of the parameter combinations maximizes our Residue Similarity Index metric. RSI evaluates the similarity of cells within a cluster (intra‐cluster similarity) compared to their similarity with cells outside that cluster.^[^ ^33^ ^]^ One could iteratively refine the connectivity weightings more precisely to this same end, though at great computational cost. This cost was considered as being prohibitive compared to its potential benefit, and so we perform only the partial parameter search. MCIST allows the simplified parameter search to be carried out automatically and can conveniently store the embeddings and spatial domains for further analysis. More information regarding this process can be found in Sections S3 and S4 (Supporting Information).

## Conflict of Interest

The authors declare no conflict of interest.

## Author Contributions

S.C. contributed to the conceptualization, methodology, data curation, data analysis, writing, and editing of the manuscript. G.W.W. was involved in the conceptualization, supervision, funding acquisition, methodology, and editing.

## Supporting information

Supporting Information

## Data Availability

The data that support the findings of this study are openly available in MCIST at https://github.com/seanfcottrell/MCIST/tree/main , reference number 1.
