# Supplementary material for: Multiscale Cell–Cell Interactive Spatial Transcriptomics Analysis
Source: Adv Sci (Weinh). 2025 Sep 15;12(44):e08358. doi: 10.1002/advs.202508358 (PMC12667549; doi:10.1002/advs.202508358)
Supplement: Supplementary file 1 — Supporting Information [file ADVS-12-e08358-s001.pdf]

Supplementary Information for Multiscale Cell-Cell Interactive Spatial Transcriptomics Analysis

Sean Cottrell[1,2] and Guo-Wei Wei[1,3,4*]

[1] Department of Mathematics,
Michigan State University, East Lansing, MI 48824, USA.
[2] Department of Computational Mathematics, Science, and Engineering,
Michigan State University, East Lansing, MI 48824, USA.
[3] Department of Electrical and Computer Engineering,
Michigan State University, East Lansing, MI 48824, USA.
[4] Department of Biochemistry and Molecular Biology,
Michigan State University, East Lansing, MI 48824, USA.

September 10, 2025

Contents

S1 Data and Preprocessing **S2**

S2 Results **S5**

S3 Parameter Values, Ensemble Learning, and Partial Optimization **S22**

S4 Residue-Similarity Index Analysis **S29**

S5 Filtration Overview **S31**

S6 Evaluating MCIST on Tumor Heterogeneity in Visium Breast Cancer Data **S32**

S7 Evaluating MCIST on Neurodevelopment Trajectory Inference **S34**

S8 Evaluation Metrics **S34**
S8.1 Adjusted Rand Index (ARI) S34
S8.2 Normalized Mutual Information (NMI) S35

*Corresponding author. Email: weig@msu.edu

S1 Data and Preprocessing

In this section, we first provide a brief summary of all 37 datasets used in this work in Table S1. These datasets involve six spatial transcriptomic technologies and are used in our work to validate the proposed multiscale cell-cell interactive spatial transcriptomics (MCIST) method.

Table S1: Spatial Transcriptomics Datasets and Statistics

Biotech	Dataset	Samples	Genes	Clusters
MERFISH [1]	04	5488	155	8
	09	5557	155	8
	14	5926	155	8
	19	5803	155	8
	24	5543	155	8
ST [2]	A1	346	15405	6
	B1	295	15109	5
	C1	176	15557	4
	D1	306	15661	4
	E1	587	15701	4
	F1	691	14861	4
	H1	613	15029	7
Visium [3]	151507	4226	33538	7
	151508	4384	33538	7
	151509	4789	33538	7
	151510	4634	33538	7
	151669	3661	33538	5
	151670	3498	33538	5
	151671	4110	33538	5
	151672	4015	33538	5
	151673	3639	33538	7
	151674	3673	33538	7
	151675	3592	33538	7
	151676	3460	33538	7
StereoSeq [4]	E9.5 E1S1	5913	25568	12
	E9.5 E2S1	5292	23756	14
	E9.5 E2S2	4356	24107	13
	E9.5 E2S3	5059	24238	13
	E9.5 E2S4	5797	23398	13
	E10.5 E1S1	18408	25201	13
	E10.5 E1S2	18647	25544	11
	E10.5 E1S3	18670	25647	16
	E10.5 E2S1	8494	22385	18
BaristaSeq [5]	Slice 1	1525	79	6
	Slice 2	2042	79	6
	Slice 3	1690	79	6
STARmap [6]	STARmap1k	1207	1020	7

Additionally, we give more detailed statistics of all 38 datasets examined in this study, including the animal of origin, the sequencing technology, the shape of the data, number of clusters, sparsity, and Max, Mean, and Median of the gene expression values. Note that one of these datasets does not have labels and thus is not used in the method validation but is studied in the Supporting Information.

For preprocessing, all datasets with more than 3000 genes were filtered to retain only the most highly variable genes using Seurat. We normalized each sample by total counts over all genes, so that every sample had the same total count after normalization, with a target value of $1e4$. The data was then log transformed. The values listed in these tables describe the summary statistics of the data prior to normalization.

Table S2: Dataset, source organism, and summary statistics for samples, genes, and cell types for 12 DLPFC datasets

Dataset	Reference	Organism	Technology	Samples	Genes	Classes	Sparsity	Max	Mean	Median
151507	[3]	Human	Visium	4226	33538	7	95.79	204	0.07	0
151508	[3]	Human	Visium	4384	33538	7	96.44	232	0.06	0
151509	[3]	Human	Visium	4789	33538	7	95.72	200	0.07	0
151510	[3]	Human	Visium	4634	33538	7	95.91	214	0.07	0
151669	[3]	Human	Visium	3661	33538	5	94.63	409	0.11	0
151670	[3]	Human	Visium	3498	33538	5	95.00	428	0.10	0
151671	[3]	Human	Visium	4110	33538	5	94.45	442	0.11	0
151672	[3]	Human	Visium	4015	33538	5	94.75	367	0.10	0
151673	[3]	Human	Visium	3639	33538	7	93.42	610	0.13	0
151674	[3]	Human	Visium	3673	33538	7	91.95	511	0.17	0
151675	[3]	Human	Visium	3592	33538	7	94.61	417	0.10	0
151676	[3]	Human	Visium	3460	33538	7	94.22	413	0.11	0

Table S3: Dataset, source organism, and summary statistics for samples, genes, and cell types for 7 HER2 Positive Breast Tumor datasets

Dataset	Reference	Organism	Technology	Samples	Genes	Classes	Sparsity	Max	Mean	Median
A1	[2]	Human	ST	346	15045	6	84.63	232	0.298	0
B1	[2]	Human	ST	295	15109	5	89.33	325	0.237	0
C1	[2]	Human	ST	176	15557	4	76.28	360	0.606	0
D1	[2]	Human	ST	306	15661	4	77.33	465	0.529	0
E1	[2]	Human	ST	587	15701	4	93.23	362	0.141	0
F1	[2]	Human	ST	691	14861	4	86.92	286	0.239	0
H1	[2]	Human	ST	613	15029	7	93.40	373	0.118	0

Table S4: Dataset, source organism, and summary statistics for samples, genes, and cell types for 5 MERFISH datasets

Dataset	Reference	Organism	Technology	Samples	Genes	Classes	Sparsity	Max	Mean	Median
04	[1]	Mouse	MERFISH	5488	155	8	58.12	188.97	1.55	0.0
09	[1]	Mouse	MERFISH	5557	155	8	56.84	181.05	1.65	0.0
14	[1]	Mouse	MERFISH	5926	155	8	57.49	197.74	1.56	0.0
19	[1]	Mouse	MERFISH	5803	155	8	57.77	223.54	1.62	0.0
24	[1]	Mouse	MERFISH	5543	155	8	58.68	227.29	1.56	0.0

Table S5: Dataset, source organism, and summary statistics for samples, genes, and cell types for 3 BaristaSeq datasets

Dataset	Reference	Organism	Technology	Samples	Genes	Classes	Sparsity	Max	Mean	Median
Slice 1	[5]	Mouse	BaristaSeq	1525	79	6	80.94	62	0.71	0.0
Slice 2	[5]	Mouse	BaristaSeq	2042	79	6	81.84	59	0.63	0.0
Slice 3	[5]	Mouse	BaristaSeq	1690	79	6	85.62	35	0.45	0.0

Table S6: Dataset, source organism, and summary statistics for samples, genes, and cell types for 9 StereoSeq datasets

Dataset	Reference	Organism	Technology	Samples	Genes	Classes	Sparsity	Max	Mean	Median
9.5 E1S1	[4]	Mouse	StereoSeq	5913	25568	12	86.02	7019	0.51	0.0
9.5 E2S1	[4]	Mouse	StereoSeq	5292	23756	14	85.62	3373	0.47	0.0
9.5 E2S2	[4]	Mouse	StereoSeq	4356	24107	13	87.08	2953	0.43	0.0
9.5 E2S3	[4]	Mouse	StereoSeq	5059	24238	13	86.55	2522	0.46	0.0
9.5 E2S4	[4]	Mouse	StereoSeq	5797	23398	13	90.00	1247	0.28	0.0
10.5 E1S1	[4]	Mouse	StereoSeq	18408	25201	13	85.78	3832	0.47	0.0
10.5 E1S2	[4]	Mouse	StereoSeq	18647	25544	11	90.48	2272	0.26	0.0
10.5 E1S3	[4]	Mouse	StereoSeq	18670	25647	16	98.00	3711	0.42	0.0
10.5 E2S1	[4]	Mouse	StereoSeq	8494	22385	18	98.00	1933	0.31	0.0

Table S7: Dataset, source organism, and summary statistics for samples, genes, and cell types Visium Breast Tumor and STARmap datasets

Dataset	Reference	Organism	Technology	Samples	Genes	Classes	Sparsity	Max	Mean	Median
STARmap	[6]	Mouse	STARmap	1207	1020	7	76.86	205	0.45	0.0
Breast Tumor	[7]	Human	Visium	3798	36601	20	84.63	6108	0.59	0.0

S2 Results

In the following tables we provide the results obtained in this study. Tables are organized according to the study from which each dataset was obtained. Table S8 lists the NMI scores for each tested method on the Visium Dorsolateral Prefrontal Cortex data [3]. Maynard et al. used the 10x Genomics Visium platform to define the spatial topography of gene expression in the six-layered human dorsolateral prefrontal cortex (DLPFC). They presented 12 datasets with an average of 3973 samples and 33538 genes.

Table S8: NMI Scores by Dataset and Method on Visium

Dataset	Method	NMI
151507	MCIST GraphST Mclust	0.7044
151508	MCIST GraphST Mclust	0.6453
151509	MCIST GraphST Mclust	0.6765
151510	MCIST GraphST Mclust	0.6529
151669	MCIST GraphST Mclust	0.6209
151670	MCIST GraphST Mclust	0.5917
151671	MCIST GraphST Mclust	0.6708
151672	MCIST GraphST Mclust	0.7016
151673	MCIST GraphST Mclust	0.7362
151674	MCIST GraphST Mclust	0.5258
151675	MCIST GraphST Mclust	0.7109
151676	MCIST GraphST Mclust	0.6776
151507	MCIST GraphST Leiden	0.6091
151508	MCIST GraphST Leiden	0.5841
151509	MCIST GraphST Leiden	0.5187
151510	MCIST GraphST Leiden	0.5493
151669	MCIST GraphST Leiden	0.6106
151670	MCIST GraphST Leiden	0.5382
151671	MCIST GraphST Leiden	0.6564
151672	MCIST GraphST Leiden	0.6670
151673	MCIST GraphST Leiden	0.6266
151674	MCIST GraphST Leiden	0.4537
151675	MCIST GraphST Leiden	0.5601
151676	MCIST GraphST Leiden	0.5055
151507	MCIST GATE Mclust	0.7121
151508	MCIST GATE Mclust	0.6743
151509	MCIST GATE Mclust	0.6686
151510	MCIST GATE Mclust	0.6612
151669	MCIST GATE Mclust	0.6237
151670	MCIST GATE Mclust	0.5721
151671	MCIST GATE Mclust	0.7074
151672	MCIST GATE Mclust	0.6840
151673	MCIST GATE Mclust	0.7157
151674	MCIST GATE Mclust	0.5396
151675	MCIST GATE Mclust	0.6649
151676	MCIST GATE Mclust	0.6681
151507	MCIST GATE Leiden	0.4830

Continued on next page

Table S8: NMI Scores by Dataset and Method on Visium

Dataset	Method	NMI
151508	MCIST GATE Leiden	0.5161
151509	MCIST GATE Leiden	0.4560
151510	MCIST GATE Leiden	0.5132
151669	MCIST GATE Leiden	0.4463
151670	MCIST GATE Leiden	0.4229
151671	MCIST GATE Leiden	0.3831
151672	MCIST GATE Leiden	0.5154
151673	MCIST GATE Leiden	0.4366
151674	MCIST GATE Leiden	0.4446
151675	MCIST GATE Leiden	0.4326
151676	MCIST GATE Leiden	0.4800
151507	MCIST SpaceFlow Mclust	0.5492
151508	MCIST SpaceFlow Mclust	0.5046
151509	MCIST SpaceFlow Mclust	0.5160
151510	MCIST SpaceFlow Mclust	0.5113
151669	MCIST SpaceFlow Mclust	0.4455
151670	MCIST SpaceFlow Mclust	0.4139
151671	MCIST SpaceFlow Mclust	0.5178
151672	MCIST SpaceFlow Mclust	0.5199
151673	MCIST SpaceFlow Mclust	0.5735
151674	MCIST SpaceFlow Mclust	0.4208
151675	MCIST SpaceFlow Mclust	0.4981
151676	MCIST SpaceFlow Mclust	0.4573
151507	MCIST SpaceFlow Leiden	0.5439
151508	MCIST SpaceFlow Leiden	0.4950
151509	MCIST SpaceFlow Leiden	0.5268
151510	MCIST SpaceFlow Leiden	0.4957
151669	MCIST SpaceFlow Leiden	0.3542
151670	MCIST SpaceFlow Leiden	0.3638
151671	MCIST SpaceFlow Leiden	0.5757
151672	MCIST SpaceFlow Leiden	0.4593
151673	MCIST SpaceFlow Leiden	0.5629
151674	MCIST SpaceFlow Leiden	0.3958
151675	MCIST SpaceFlow Leiden	0.4850
151676	MCIST SpaceFlow Leiden	0.4562
151670	SpaGCN	0.4411
151671	SpaGCN	0.6233
151676	SpaGCN	0.4940
151508	SpaGCN	0.5396
151509	SpaGCN	0.5281
151674	SpaGCN	0.4989
151510	SpaGCN	0.5804
151507	SpaGCN	0.5508
151675	SpaGCN	0.5338

Continued on next page

Table S8: NMI Scores by Dataset and Method on Visium

Dataset	Method	NMI
151672	SpaGCN	0.6502
151669	SpaGCN	0.4559
151673	SpaGCN	0.4914
151671	SEDR	0.6614
151670	SEDR	0.5196
151509	SEDR	0.5763
151676	SEDR	0.4984
151508	SEDR	0.4804
151507	SEDR	0.4879
151675	SEDR	0.5326
151674	SEDR	0.5686
151510	SEDR	0.5348
151669	SEDR	0.5960
151672	SEDR	0.4710
151673	SEDR	0.6315
151674	BASS	0.5786
151510	BASS	0.5713
151507	BASS	0.6532
151675	BASS	0.6071
151672	BASS	0.6521
151669	BASS	0.5926
151670	BASS	0.4571
151671	BASS	0.6815
151508	BASS	0.6169
151676	BASS	0.5571
151509	BASS	0.6269
151673	BASS	0.7095
151510	conST	0.4935
151674	conST	0.6013
151675	conST	0.4023
151507	conST	0.5052
151672	conST	0.4212
151669	conST	0.5142
151670	conST	0.5468
151671	conST	0.6238
151676	conST	0.5426
151508	conST	0.4611
151509	conST	0.5397
151673	conST	0.6716
151509	SpaceFlow	0.5182
151508	SpaceFlow	0.5243
151676	SpaceFlow	0.4036
151671	SpaceFlow	0.4602
151670	SpaceFlow	0.3818

Continued on next page

Table S8: NMI Scores by Dataset and Method on Visium

Dataset	Method	NMI
151669	SpaceFlow	0.4559
151672	SpaceFlow	0.5412
151675	SpaceFlow	0.4665
151507	SpaceFlow	0.6477
151510	SpaceFlow	0.5682
151674	SpaceFlow	0.4353
151673	SpaceFlow	0.4466
151672	Louvain	0.2263
151669	Louvain	0.1613
151674	Louvain	0.3352
151510	Louvain	0.2405
151507	Louvain	0.2149
151675	Louvain	0.3533
151676	Louvain	0.3189
151508	Louvain	0.2222
151509	Louvain	0.3402
151670	Louvain	0.1619
151671	Louvain	0.2385
151673	Louvain	0.3026
151670	SCAN-IT	0.5144
151671	SCAN-IT	0.5359
151676	SCAN-IT	0.5690
151508	SCAN-IT	0.6170
151509	SCAN-IT	0.6781
151674	SCAN-IT	0.5400
151510	SCAN-IT	0.6200
151507	SCAN-IT	0.6702
151675	SCAN-IT	0.5223
151672	SCAN-IT	0.6154
151669	SCAN-IT	0.3588
151673	SCAN-IT	0.5765
151676	stLearn	0.5389
151508	stLearn	0.5175
151509	stLearn	0.6081
151670	stLearn	0.3526
151671	stLearn	0.5799
151672	stLearn	0.4878
151669	stLearn	0.5145
151674	stLearn	0.5469
151510	stLearn	0.4987
151507	stLearn	0.6138
151675	stLearn	0.5705
151673	stLearn	0.5477
151510	CCST	0.4839

Continued on next page

Table S8: NMI Scores by Dataset and Method on Visium

Dataset	Method	NMI
151674	CCST	0.4918
151675	CCST	0.4749
151507	CCST	0.5952
151672	CCST	0.5777
151669	CCST	0.4216
151670	CCST	0.4304
151671	CCST	0.5544
151676	CCST	0.5366
151508	CCST	0.5437
151509	CCST	0.5619
151673	CCST	0.4489
151507	BayesSpace	0.6252
151675	BayesSpace	0.6836
151674	BayesSpace	0.4820
151510	BayesSpace	0.5625
151669	BayesSpace	0.6096
151672	BayesSpace	0.5963
151671	BayesSpace	0.6852
151670	BayesSpace	0.5547
151509	BayesSpace	0.5964
151676	BayesSpace	0.5298
151508	BayesSpace	0.5994
151673	BayesSpace	0.6878
151670	STAGATE	0.3955
151671	STAGATE	0.4538
151676	STAGATE	0.4573
151508	STAGATE	0.4811
151509	STAGATE	0.5333
151510	STAGATE	0.4935
151674	STAGATE	0.5225
151675	STAGATE	0.5638
151507	STAGATE	0.5357
151672	STAGATE	0.5061
151669	STAGATE	0.3596
151673	STAGATE	0.5924
151672	Leiden	0.2163
151669	Leiden	0.1575
151674	Leiden	0.3231
151510	Leiden	0.2507
151507	Leiden	0.2160
151675	Leiden	0.3423
151508	Leiden	0.2265
151676	Leiden	0.3224
151509	Leiden	0.2907

Continued on next page

Table S8: NMI Scores by Dataset and Method on Visium

Dataset	Method	NMI
151670	Leiden	0.1645
151671	Leiden	0.2405
151673	Leiden	0.3075
151507	GraphST	0.6862
151508	GraphST	0.5634
151509	GraphST	0.6298
151510	GraphST	0.5873
151669	GraphST	0.5913
151670	GraphST	0.4742
151671	GraphST	0.6825
151672	GraphST	0.6670
151673	GraphST	0.6975
151674	GraphST	0.5257
151675	GraphST	0.6389
151676	GraphST	0.6122

In Andersonn et al., the authors investigate spatial gene expression in HER2-positive breast tumors using spatial transcriptomics technology [2]. They provide 7 datasets with ground truth annotations, with 431 samples and 15280 genes on average. In Table S9 we provide the NMI scores for each methods across these 7 datasets.

Table S9: NMI Scores by Dataset and Method on ST

Dataset	Method	NMI
A1	MCIST GraphST Mclust	0.2505
B1	MCIST GraphST Mclust	0.3291
C1	MCIST GraphST Mclust	0.1876
D1	MCIST GraphST Mclust	0.2745
E1	MCIST GraphST Mclust	0.2452
F1	MCIST GraphST Mclust	0.0996
H1	MCIST GraphST Mclust	0.4526
A1	MCIST GraphST Leiden	0.2528
B1	MCIST GraphST Leiden	0.4207
C1	MCIST GraphST Leiden	0.0642
D1	MCIST GraphST Leiden	0.2464
E1	MCIST GraphST Leiden	0.1730
F1	MCIST GraphST Leiden	0.1400
H1	MCIST GraphST Leiden	0.4397
A1	MCIST GATE Mclust	0.2060
B1	MCIST GATE Mclust	0.3309
C1	MCIST GATE Mclust	0.1971
D1	MCIST GATE Mclust	0.1903

Continued on next page

Table S9: NMI Scores by Dataset and Method on ST

Dataset	Method	NMI
E1	MCIST GATE Mclust	0.2740
F1	MCIST GATE Mclust	0.1157
H1	MCIST GATE Mclust	0.4819
A1	MCIST GATE Leiden	0.2056
B1	MCIST GATE Leiden	0.3334
C1	MCIST GATE Leiden	0.1124
D1	MCIST GATE Leiden	0.1293
E1	MCIST GATE Leiden	0.2865
F1	MCIST GATE Leiden	0.1116
H1	MCIST GATE Leiden	0.4200
A1	MCIST SpaceFlow Mclust	0.3053
B1	MCIST SpaceFlow Mclust	0.3979
C1	MCIST SpaceFlow Mclust	0.3040
D1	MCIST SpaceFlow Mclust	0.2943
E1	MCIST SpaceFlow Mclust	0.2056
F1	MCIST SpaceFlow Mclust	0.1446
H1	MCIST SpaceFlow Mclust	0.4317
A1	MCIST SpaceFlow Leiden	0.2640
B1	MCIST SpaceFlow Leiden	0.4489
C1	MCIST SpaceFlow Leiden	0.2130
D1	MCIST SpaceFlow Leiden	0.2083
E1	MCIST SpaceFlow Leiden	0.2097
F1	MCIST SpaceFlow Leiden	0.1309
H1	MCIST SpaceFlow Leiden	0.4000
C1	STAGATE	0.0692
B1	STAGATE	0.1698
A1	STAGATE	0.0355
D1	STAGATE	0.0471
E1	STAGATE	0.1710
H1	STAGATE	0.3160
F1	STAGATE	0.0388
A1	SpaGCN	0.0963
B1	SpaGCN	0.2619
C1	SpaGCN	-0.0138
D1	SpaGCN	0.2434
E1	SpaGCN	0.0237
F1	SpaGCN	0.0751
H1	SpaGCN	0.3147
A1	Louvain	0.0636
B1	Louvain	0.2267
C1	Louvain	-0.0232
D1	Louvain	0.1757
E1	Louvain	0.0558
F1	Louvain	0.0561

Continued on next page

Table S9: NMI Scores by Dataset and Method on ST

Dataset	Method	NMI
H1	Louvain	0.2436
A1	BayesSpace	0.3521
B1	BayesSpace	0.2451
C1	BayesSpace	0.1186
D1	BayesSpace	0.1154
E1	BayesSpace	0.0229
F1	BayesSpace	0.0976
H1	BayesSpace	0.3450
A1	GraphST	0.0908
B1	GraphST	0.3127
C1	GraphST	-0.0122
D1	GraphST	0.1217
E1	GraphST	0.0703
F1	GraphST	0.1342
H1	GraphST	0.3647
A1	SEDR	0.1495
B1	SEDR	0.2624
C1	SEDR	0.0576
D1	SEDR	0.1980
E1	SEDR	0.0474
F1	SEDR	0.1224
H1	SEDR	0.3439
A1	SCAN-IT	0.0566
B1	SCAN-IT	0.4050
C1	SCAN-IT	0.2549
D1	SCAN-IT	0.1183
E1	SCAN-IT	0.1049
F1	SCAN-IT	0.0256
H1	SCAN-IT	0.3016
A1	BASS	0.4499
B1	BASS	0.3401
C1	BASS	0.0561
D1	BASS	0.1965
E1	BASS	0.0236
F1	BASS	0.0772
H1	BASS	0.2715
A1	SPACEFLOW	0.0424
B1	SPACEFLOW	0.3382
C1	SPACEFLOW	0.1218
D1	SPACEFLOW	0.1322
E1	SPACEFLOW	0.0974
F1	SPACEFLOW	0.0159
H1	SPACEFLOW	0.2674
A1	Leiden	0.0556

Continued on next page

Table S9: NMI Scores by Dataset and Method on ST

Dataset	Method	NMI
B1	Leiden	0.2914
C1	Leiden	0.1431
D1	Leiden	0.1995
E1	Leiden	0.0538
F1	Leiden	0.1103
H1	Leiden	0.1091

In Table S10 we provide the NMI scores for each method tested on the MERFISH data. Multiplexed error-robust fluorescence in situ hybridization (MERFISH) allows simultaneous imaging of numerous RNA species in their native cellular environment and hence spatially resolved single-cell transcriptomic measurements [1]. We validated our method on 5 datasets, with 5663 samples and 155 genes on average. Each dataset is obtained from the mouse preoptic hypothalamus.

Table S10: NMI Scores by Dataset and Method on MERFISH

Dataset	Method	NMI
04	MCIST GraphST Mclust	0.2485
09	MCIST GraphST Mclust	0.3899
14	MCIST GraphST Mclust	0.4193
19	MCIST GraphST Mclust	0.4663
24	MCIST GraphST Mclust	0.4714
04	MCIST GraphST Leiden	0.4281
09	MCIST GraphST Leiden	0.5112
14	MCIST GraphST Leiden	0.4785
19	MCIST GraphST Leiden	0.5761
24	MCIST GraphST Leiden	0.5510
04	MCIST GATE Mclust	0.4766
09	MCIST GATE Mclust	0.5154
14	MCIST GATE Mclust	0.5001
19	MCIST GATE Mclust	0.6374
24	MCIST GATE Mclust	0.5331
04	MCIST GATE Leiden	0.4931
09	MCIST GATE Leiden	0.5832
14	MCIST GATE Leiden	0.5426
19	MCIST GATE Leiden	0.5767
24	MCIST GATE Leiden	0.6303
04	MCIST SpaceFlow Mclust	0.4301
09	MCIST SpaceFlow Mclust	0.5630
14	MCIST SpaceFlow Mclust	0.4708
19	MCIST SpaceFlow Mclust	0.6188
24	MCIST SpaceFlow Mclust	0.6044
04	MCIST SpaceFlow Leiden	0.5531

Continued on next page

Table S10: NMI Scores by Dataset and Method on MERFISH

Dataset	Method	NMI
09	MCIST SpaceFlow Leiden	0.5844
14	MCIST SpaceFlow Leiden	0.5423
19	MCIST SpaceFlow Leiden	0.5605
24	MCIST SpaceFlow Leiden	0.5898
09	SpaGCN	0.2253
04	SpaGCN	0.1850
24	SpaGCN	0.2266
19	SpaGCN	0.2164
14	SpaGCN	0.2171
04	SEDR	0.0924
24	SEDR	0.1605
09	SEDR	0.1396
14	SEDR	0.2143
19	SEDR	0.0954
09	BASS	0.5020
04	BASS	0.4273
24	BASS	0.5729
19	BASS	0.5664
14	BASS	0.5260
14	conST	0.1188
19	conST	0.1182
04	conST	0.1018
24	conST	0.1171
09	conST	0.0918
24	SpaceFlow	0.5854
04	SpaceFlow	0.3423
09	SpaceFlow	0.5949
14	SpaceFlow	0.4664
19	SpaceFlow	0.5760
04	Louvain	0.1525
24	Louvain	0.1749
09	Louvain	0.1676
14	Louvain	0.1747
19	Louvain	0.1745
14	SCAN-IT	0.5535
19	SCAN-IT	0.5909
04	SCAN-IT	0.5480
24	SCAN-IT	0.6300
09	SCAN-IT	0.5602
09	CCST	0.4496
24	CCST	0.5021
04	CCST	0.4534
19	CCST	0.4600
14	CCST	0.4617

Continued on next page

Table S10: NMI Scores by Dataset and Method on MERFISH

Dataset	Method	NMI
04	STAGATE	0.0839
24	STAGATE	0.1415
09	STAGATE	0.2693
14	STAGATE	0.2713
19	STAGATE	0.1897
09	Leiden	0.1832
04	Leiden	0.1820
24	Leiden	0.1763
19	Leiden	0.1738
14	Leiden	0.1720
04	GraphST	0.2079
09	GraphST	0.3542
14	GraphST	0.3542
19	GraphST	0.3268
24	GraphST	0.3440

In Table S11, we present the results for each method tested on 7 StereoSeq datasets. StereoSeq combines DNA nanoball (DNB)-patterned arrays and in situ RNA capture to create spatial enhanced resolution omics-sequencing. Chen at al. applied Stereo-seq to generate the mouse organogenesis spatiotemporal transcriptomic atlas (MOSTA), which maps with single-cell resolution and high sensitivity the kinetics and directionality of transcriptional variation during mouse organogenesis [4]. The 7 chosen datasets have 9067 samples and 24544 genes on average.

Table S11: NMI Scores by Dataset and Method on StereoSeq

Dataset	Method	NMI
E10.5 E1S1	MCIST GraphST Mclust	0.5340
E10.5 E1S2	MCIST GraphST Mclust	0.4522
E9.5 E1S1	MCIST GraphST Mclust	0.5472
E9.5 E2S1	MCIST GraphST Mclust	0.5460
E9.5 E2S2	MCIST GraphST Mclust	0.5765
E9.5 E2S3	MCIST GraphST Mclust	0.6278
E9.5 E2S4	MCIST GraphST Mclust	0.5680
E10.5 E1S1	MCIST GraphST Leiden	0.5572
E10.5 E1S2	MCIST GraphST Leiden	0.4798
E9.5 E1S1	MCIST GraphST Leiden	0.5569
E9.5 E2S1	MCIST GraphST Leiden	0.5731
E9.5 E2S2	MCIST GraphST Leiden	0.6021
E9.5 E2S3	MCIST GraphST Leiden	0.6235
E9.5 E2S4	MCIST GraphST Leiden	0.6162
E10.5 E1S1	MCIST GATE Mclust	0.5596
E10.5 E1S2	MCIST GATE Mclust	0.4637

Continued on next page

Table S11: NMI Scores by Dataset and Method on StereoSeq

Dataset	Method	NMI
E9.5 E1S1	MCIST GATE Mclust	0.5735
E9.5 E2S1	MCIST GATE Mclust	0.6108
E9.5 E2S2	MCIST GATE Mclust	0.5980
E9.5 E2S3	MCIST GATE Mclust	0.5992
E9.5 E2S4	MCIST GATE Mclust	0.5756
E10.5 E1S1	MCIST GATE Leiden	0.5703
E10.5 E1S2	MCIST GATE Leiden	0.4832
E9.5 E1S1	MCIST GATE Leiden	0.5584
E9.5 E2S1	MCIST GATE Leiden	0.5930
E9.5 E2S2	MCIST GATE Leiden	0.5534
E9.5 E2S3	MCIST GATE Leiden	0.6076
E9.5 E2S4	MCIST GATE Leiden	0.5264
E10.5 E1S1	MCIST SpaceFlow Mclust	0.5360
E10.5 E1S2	MCIST SpaceFlow Mclust	0.4357
E9.5 E1S1	MCIST SpaceFlow Mclust	0.5356
E9.5 E2S1	MCIST SpaceFlow Mclust	0.4851
E9.5 E2S2	MCIST SpaceFlow Mclust	0.6021
E9.5 E2S3	MCIST SpaceFlow Mclust	0.5916
E9.5 E2S4	MCIST SpaceFlow Mclust	0.5171
E10.5 E1S1	MCIST SpaceFlow Leiden	0.5549
E10.5 E1S2	MCIST SpaceFlow Leiden	0.4221
E9.5 E1S1	MCIST SpaceFlow Leiden	0.5456
E9.5 E2S1	MCIST SpaceFlow Leiden	0.5133
E9.5 E2S2	MCIST SpaceFlow Leiden	0.5770
E9.5 E2S3	MCIST SpaceFlow Leiden	0.6090
E9.5 E2S4	MCIST SpaceFlow Leiden	0.5299
E10.5 E1S1	BASS	0.4688
E10.5 E1S2	BASS	0.3931
E9.5 E1S1	BASS	0.4433
E9.5 E2S1	BASS	0.5336
E9.5 E2S2	BASS	0.5838
E9.5 E2S3	BASS	0.6237
E9.5 E2S4	BASS	0.5526
E9.5 E1S1	conST	0.4906
E9.5 E2S1	conST	0.4875
E9.5 E2S3	conST	0.5776
E9.5 E2S4	conST	0.4829
E9.5 E1S1	GraphST	0.5358
E9.5 E2S1	GraphST	0.5506
E9.5 E2S2	GraphST	0.5822
E9.5 E2S3	GraphST	0.6162
E9.5 E2S4	GraphST	0.5767
E10.5 E1S1	GraphST	0.5040
E10.5 E1S2	GraphST	0.4210

Continued on next page

Table S11: NMI Scores by Dataset and Method on StereoSeq

Dataset	Method	NMI
E10.5 E1S1	Leiden	0.3674
E10.5 E1S2	Leiden	0.2266
E9.5 E1S1	Leiden	0.2664
E9.5 E2S1	Leiden	0.3363
E9.5 E2S2	Leiden	0.3128
E9.5 E2S3	Leiden	0.3693
E9.5 E2S4	Leiden	0.3909
E10.5 E1S1	Louvain	0.3545
E10.5 E1S2	Louvain	0.2336
E9.5 E1S1	Louvain	0.2801
E9.5 E2S1	Louvain	0.3113
E9.5 E2S2	Louvain	0.2693
E9.5 E2S3	Louvain	0.3663
E9.5 E2S4	Louvain	0.3960
E10.5 E1S1	SCAN-IT	0.4414
E10.5 E1S2	SCAN-IT	0.4031
E9.5 E1S1	SCAN-IT	0.5036
E9.5 E2S1	SCAN-IT	0.5083
E9.5 E2S2	SCAN-IT	0.5007
E9.5 E2S3	SCAN-IT	0.5576
E9.5 E2S4	SCAN-IT	0.5073
E10.5 E1S1	SEDR	0.4868
E10.5 E1S2	SEDR	0.4256
E9.5 E1S1	SEDR	0.5113
E9.5 E2S1	SEDR	0.5349
E9.5 E2S2	SEDR	0.5709
E9.5 E2S3	SEDR	0.6852
E9.5 E2S4	SEDR	0.5372
E10.5 E1S1	SpaceFlow	0.3033
E10.5 E1S2	SpaceFlow	0.4185
E9.5 E1S1	SpaceFlow	0.4820
E9.5 E2S1	SpaceFlow	0.2220
E9.5 E2S2	SpaceFlow	0.4640
E9.5 E2S3	SpaceFlow	0.3291
E9.5 E2S4	SpaceFlow	0.2525
E10.5 E1S1	SpaGCN	0.4380
E10.5 E1S2	SpaGCN	0.2474
E9.5 E1S1	SpaGCN	0.3339
E9.5 E2S1	SpaGCN	0.4105
E9.5 E2S2	SpaGCN	0.4256
E9.5 E2S3	SpaGCN	0.5124
E9.5 E2S4	SpaGCN	0.4466
E10.5 E1S1	STAGATE	0.4231
E10.5 E1S2	STAGATE	0.5060

Continued on next page

Table S11: NMI Scores by Dataset and Method on StereoSeq

Dataset	Method	NMI
E9.5 E1S1	STAGATE	0.5045
E9.5 E2S1	STAGATE	0.5208
E9.5 E2S2	STAGATE	0.5025
E9.5 E2S3	STAGATE	0.5177
E9.5 E2S4	STAGATE	0.5658

Beyond these seven datasets, there are two additional datasets which were tested on only a subset of the methods in the latest benchmarking study. We note in Table S12 that MCIST was able to outperform or perform competitively with these methods on this data as well.

Table S12: NMI Scores by Dataset and Method on StereoSeq2

Dataset	Method	NMI
E10.5 E1S3	MCIST GraphST Mclust	0.5685
E10.5 E2S1	MCIST GraphST Mclust	0.6226
E10.5 E1S3	MCIST GraphST Leiden	0.5547
E10.5 E2S1	MCIST GraphST Leiden	0.5909
E10.5 E1S3	MCIST GATE Mclust	0.5618
E10.5 E2S1	MCIST GATE Mclust	0.6005
E10.5 E1S3	MCIST GATE Leiden	0.5370
E10.5 E2S1	MCIST GATE Leiden	0.5903
E10.5 E1S3	MCIST SpaceFlow Mclust	0.4501
E10.5 E2S1	MCIST SpaceFlow Mclust	0.5782
E10.5 E1S3	MCIST SpaceFlow Leiden	0.4809
E10.5 E2S1	MCIST SpaceFlow Leiden	0.5747
E10.5 E1S3	GraphST	0.5370
E10.5 E1S3	BASS	0.5366
E10.5 E2S1	conST	0.5220
E10.5 E1S3	Louvain	0.2914
E10.5 E2S1	Louvain	0.2973
E10.5 E1S3	Leiden	0.3332
E10.5 E2S1	Leiden	0.3253
E10.5 E1S3	SpaGCN	0.5439
E10.5 E2S1	SpaGCN	0.6415
E10.5 E1S3	SpaceFlow	0.3175
E10.5 E2S1	SpaceFlow	0.3506
E10.5 E1S3	SCAN-IT	0.4369
E10.5 E2S1	SCAN-IT	0.5569
E10.5 E2S1	SEDR	0.6319
E10.5 E2S1	GraphST	0.6028

We also test our method on three slices of the BaristaSeq data [5]. In Table S13 we note the resuslts for each method on each dataset.

Table S13: NMI Scores by Dataset and Method on BaristaSeq

Dataset	Method	NMI
Slice 1	MCIST GraphST Mclust	0.4246
Slice 2	MCIST GraphST Mclust	0.5043
Slice 3	MCIST GraphST Mclust	0.2420
Slice 1	MCIST GraphST Leiden	0.5633
Slice 2	MCIST GraphST Leiden	0.6447
Slice 3	MCIST GraphST Leiden	0.4174
Slice 1	MCIST GATE Mclust	0.5309
Slice 2	MCIST GATE Mclust	0.5312
Slice 3	MCIST GATE Mclust	0.4640
Slice 1	MCIST GATE Leiden	0.6315
Slice 2	MCIST GATE Leiden	0.7624
Slice 3	MCIST GATE Leiden	0.4134
Slice 1	MCIST SpaceFlow Mclust	0.6539
Slice 2	MCIST SpaceFlow Mclust	0.7562
Slice 3	MCIST SpaceFlow Mclust	0.6360
Slice 1	MCIST SpaceFlow Leiden	0.6689
Slice 2	MCIST SpaceFlow Leiden	0.8227
Slice 3	MCIST SpaceFlow Leiden	0.6562
Slice 1	BASS	0.8162
Slice 2	BASS	0.7111
Slice 3	BASS	0.6440
Slice 1	conST	0.0349
Slice 3	conST	0.0309
Slice 2	conST	0.0397
Slice 3	SpaceFlow	0.6353
Slice 2	SpaceFlow	0.7266
Slice 1	SpaceFlow	0.7022
Slice 1	Louvain	0.0238
Slice 2	Louvain	0.1036
Slice 3	Louvain	0.0603
Slice 2	SCAN-IT	0.7208
Slice 3	SCAN-IT	0.7170
Slice 1	SCAN-IT	0.7324
Slice 2	CCST	0.6625
Slice 3	CCST	0.5699
Slice 1	CCST	0.6271
Slice 1	Leiden	0.0613
Slice 2	Leiden	0.1045
Slice 3	Leiden	0.0817
Slice 1	GraphST	0.3549
Slice 2	GraphST	0.4208
Slice 3	GraphST	0.3410
Slice 1	STAGATE	0.3362
Slice 3	STAGATE	0.2802

Continued on next page

Table S13: NMI Scores by Dataset and Method on BaristaSeq

Dataset	Method	NMI
Slice 2	STAGATE	0.4914
Slice 2	SpaGCN	0.2692
Slice 3	SpaGCN	0.1865
Slice 1	SpaGCN	0.3074
Slice 3	SEDR	0.0477
Slice 2	SEDR	0.0635
Slice 1	SEDR	0.0421

Lastly, we validated our method on the STARmap data, with 1207 samples. Table S14 provides the NMI scores of each method on the STARmap data. The STARmap data contains 1020 genes sequenced from a mouse brain at single-cell resolution with high efficiency, accuracy, and reproducibility [6].

Table S14: NMI Scores by Dataset and Method on STARmap

Dataset	Method	NMI
STARmap	MCIST GraphST Mclust	0.5200
STARmap	MCIST GraphST Leiden	0.5353
STARmap	MCIST GATE Mclust	0.5120
STARmap	MCIST GATE Leiden	0.3800
STARmap	MCIST SpaceFlow Mclust	0.7072
STARmap	MCIST SpaceFlow Leiden	0.7211
STARmap	SpaGCN	0.3704
STARmap	SEDR	0.2828
STARmap	BASS	0.6627
STARmap	conST	0.2406
STARmap	SpaceFlow	0.6618
STARmap	Louvain	0.1135
STARmap	SCAN-IT	0.7143
STARmap	CCST	0.5545
STARmap	Leiden	0.1164
STARmap	GraphST	0.5036
STARmap	STAGATE	0.3904

We note that these tables contain multiple MCIST scores for each platform, corresponding to different choices of spatial deep learning method and different clustering algorithm for use in the final Agglomerative clustering. In the main text, for Visium, we combine GraphST and Mclust clustering in MCIST. For StereoSeq, GraphST and Leiden. For MERFISH and BaristaSeq, SpaceFlow and Leiden. For ST and STARmap, SpaceFlow and Mclust. However, it is worthwhile to note how each of these pairings performs on average over all 37 datasets, as well as noting the peformance of STAGATE, the most scalable method in terms of runtime and memory usage. In Figure S1 we illustrate this comparison for each platform, and in Figure S2 we illustrate the performances averaged over all 37 datatsets. We note that when MCIST utilizes

the STAGATE deep learning method and the Mclust algorithm for consensus clustering, it is able to still achieve state-of-the-art results, outperforming BASS by 4%.

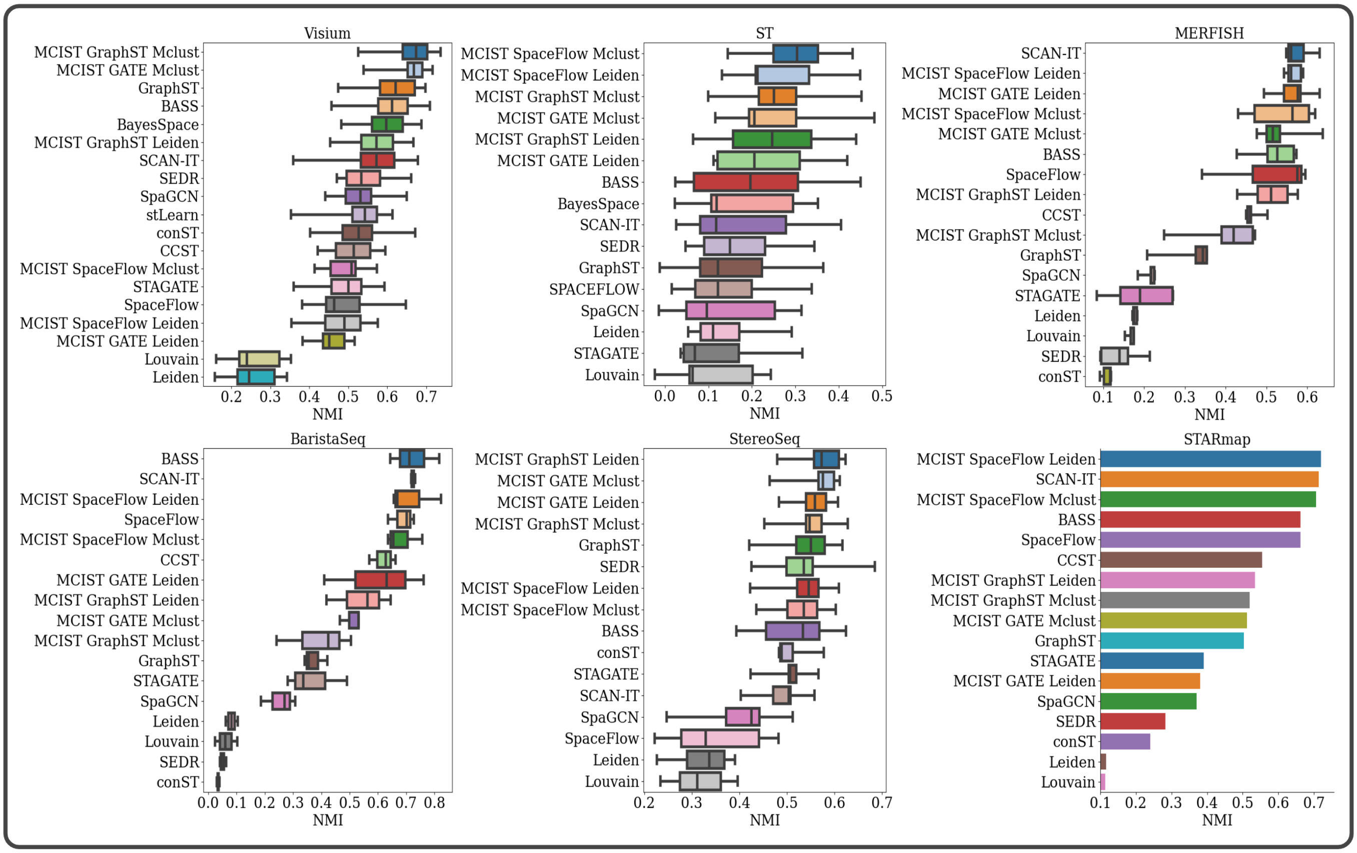

Figure S1: Comparisons of NMI scores for different MCIST versions among each ST platform.

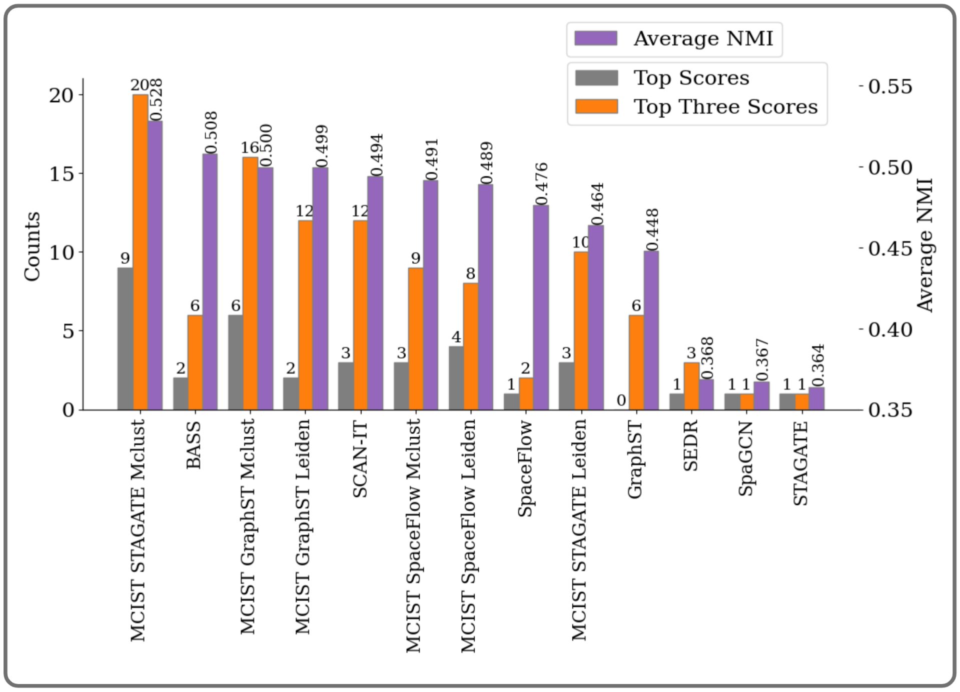

Figure S2: Comparison of NMI scores for different MCIST versions averaged over all 37 datasets.

S3 Parameter Values, Ensemble Learning, and Partial Optimization

Topological PCA is obtained via solving the following equation:

$$\min_{U,Q} \|X - UQ^T\|_{2,1} + \beta\|Q\|_{2,1} + \gamma \text{Tr}(Q^T(L_P)Q), \quad \text{s.t } Q^TQ = I_m \tag{1}$$

We note the presence of several hyper-parameters, notably β, γ, and each $\{\zeta_k\}$. Optimal performance of Topological PCA relies on the appropriate choice of these parameters. However, an extensive parameter search is time consuming, computationally costly, and difficult in the absence of ground truth annotations. We therefore found it necessary to significantly restrict the parameter space and to perform a consensus clustering using a small subset of connectivity weighting parameter combinations. Biologically, this is analogous to considering multiple sets of scales of the modeled cell interactions. This ensemble procedure is displayed in Figure S3.

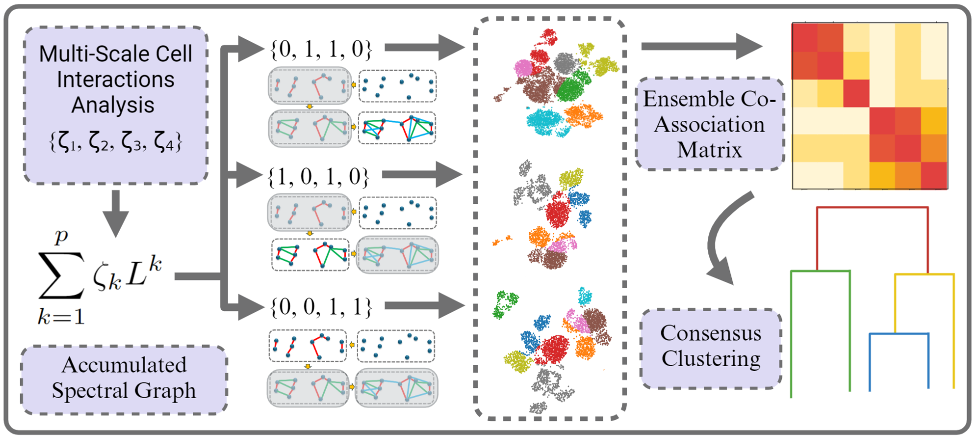

Figure S3: Overview of the Multi-Scale cell interactions analysis for ensemble clustering of Spatial Transcriptomics data. Multiple scales of cell interactions are considered and clustered before performing a final consensus clustering.

In the case where we do perform parameter optimization, we can obtain the optimal values via a Grid Search where we are seeking to maximize the Residue Similarity Index of our embedding. In Tables S15-S17 we provide some of the $\{\zeta_k\}$ values which were among 8 different tests while fixing the embedding dimensionality m, as well as the scale paramerers β, and γ. If one were to wish to make use of the MCIST embeddings for downstream analysis, such as pseudotime inference of differential gene expression, then an at least partially optimal choice of parameters becomes necessary. We display this optimization procedure in Figure S4.

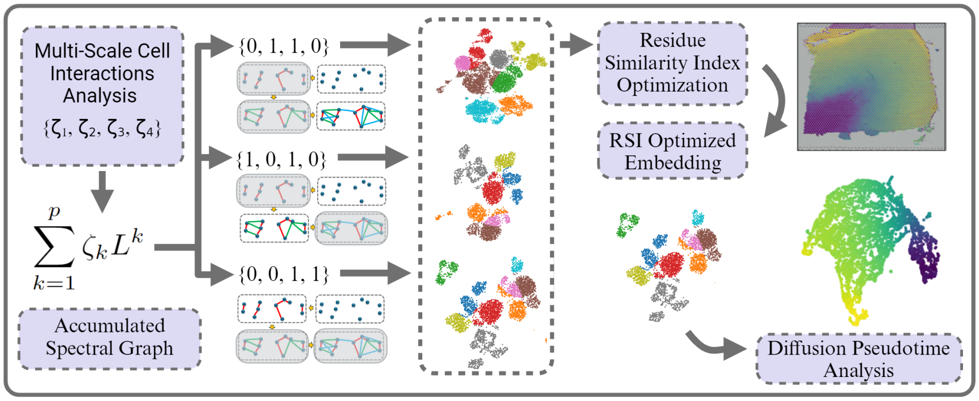

Figure S4: Overview of the Multi-Scale cell interactions analysis for RSI optimization of the cell embeddings. Multiple scales of cell interactions are considered and the one which maximizes the RSI score is utilized for downstream analysis, such as Pseudotime inference.

However, a parameter free approach is certainly preferable both for reasons of robustness as well as convenience, particularly if it can still maintain competitive performance. To this end we utilize an ensemble learning framework for MCIST. We consider the eight different $\{\zeta_k\}$ connectivity weighting configurations and produce a tPCA gene expression embedding from each. This is aligned with the output of the spatially informed encoder via a canonical correlation analysis, after which we can perform unsupervised clustering with the Mclust or Leiden algorithm. Each of these different clusterings is then used for a hierarchical / agglomerative clustering. Specifically, the clusterings are used to define a co-association matrix, $C(i, j)$, which is built based on how often pairs of cells are assigned to the same cluster across the multiple ζ configurations, and then normalized by the number of clusters. The co-association matrix then can define a distance metric for the hierarchical clustering: $D(i, j) = 1 - C(i, j)$.

In Figures S5-S7 we provide illustrations which demonstrate how the ensemble learning approach compares to the individual choices for scales of cell interaction to be preserved in tCPA. Bars indicate the performance of different $\{\zeta\}$ configurations, while the dotted line depicts the consensus clustering scores when using STAGATE and Mclust clustering in MCIST.

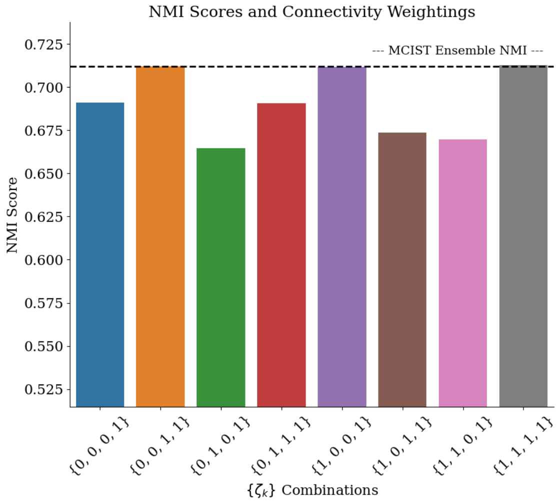

Comparison of NMI scores for different parameter combinations versus NMI score of consensus clustering on DLPFC Slice 151507

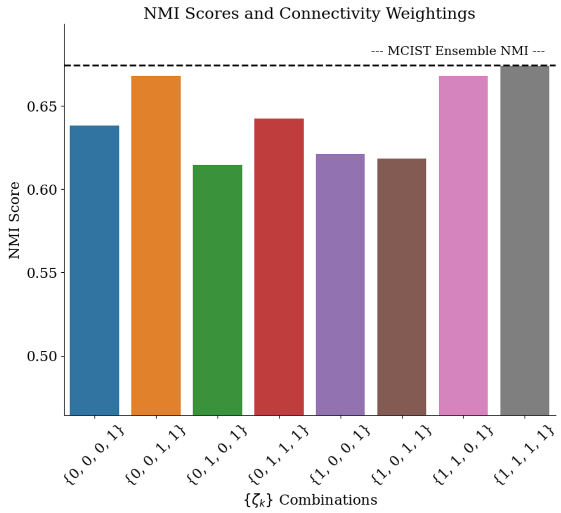

Comparison of NMI scores for different parameter combinations versus NMI score of consensus clustering on DLPFC Slice 151508

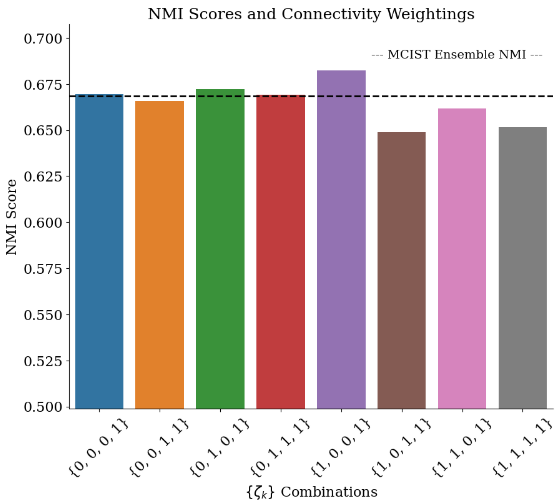

Comparison of NMI scores for different parameter combinations versus NMI score of consensus clustering on DLPFC Slice 151509

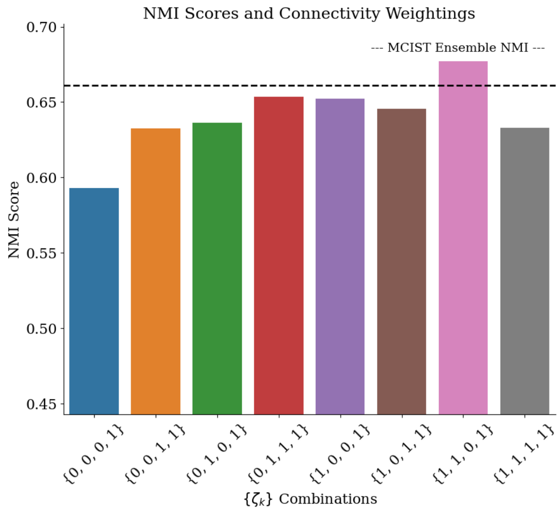

Comparison of NMI scores for different parameter combinations versus NMI score of consensus clustering on DLPFC Slice 151510

Figure S5: NMI Scores for different parameter combinations on DLPFC 151507-151510.

(a) DLPFC Slice 151507

$\{\zeta_k\}$	NMI	RSI
0, 0, 0, 1	0.6912	0.1532
0, 0, 1, 1	0.7121	0.2857
0, 1, 0, 1	0.6647	0.2729
0, 1, 1, 1	0.6907	0.4163
1, 0, 0, 1	0.7118	0.1778
1, 0, 1, 1	0.6737	0.3752
1, 1, 0, 1	0.6698	0.3536
1, 1, 1, 1	0.7128	0.4541

(b) DLPFC Slice 151508

$\{\zeta_k\}$	NMI	RSI
0, 0, 0, 1	0.6380	0.3244
0, 0, 1, 1	0.6679	0.2893
0, 1, 0, 1	0.6144	0.3393
0, 1, 1, 1	0.6424	0.3002
1, 0, 0, 1	0.6212	0.3180
1, 0, 1, 1	0.6182	0.3103
1, 1, 0, 1	0.6680	0.2938
1, 1, 1, 1	0.6741	0.3188

(c) DLPFC Slice 151509

$\{\zeta_k\}$	NMI	RSI
0, 0, 0, 1	0.6695	0.1831
0, 0, 1, 1	0.6658	0.1133
0, 1, 0, 1	0.6722	0.1127
0, 1, 1, 1	0.6693	0.0958
1, 0, 0, 1	0.6824	0.1046
1, 0, 1, 1	0.6488	0.2248
1, 1, 0, 1	0.6616	0.1117
1, 1, 1, 1	0.6516	0.2240

(d) DLPFC Slice 151510

$\{\zeta_k\}$	NMI	RSI
0, 0, 0, 1	0.5930	0.2011
0, 0, 1, 1	0.6324	0.1027
0, 1, 0, 1	0.6362	0.1354
0, 1, 1, 1	0.6537	0.2028
1, 0, 0, 1	0.6523	0.2057
1, 0, 1, 1	0.6458	0.1747
1, 1, 0, 1	0.6769	0.1707
1, 1, 1, 1	0.6330	0.2207

Table S15: NMI and RSI Scores by ζ Combinations on DLPFC Slices 151507, 151508, 151509, and 151510

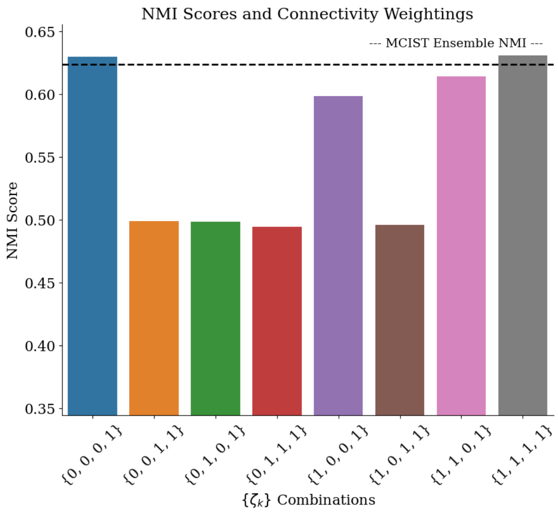

Comparison of NMI scores for different parameter combinations versus NMI score of consensus clustering on DLPFC Slice 151669

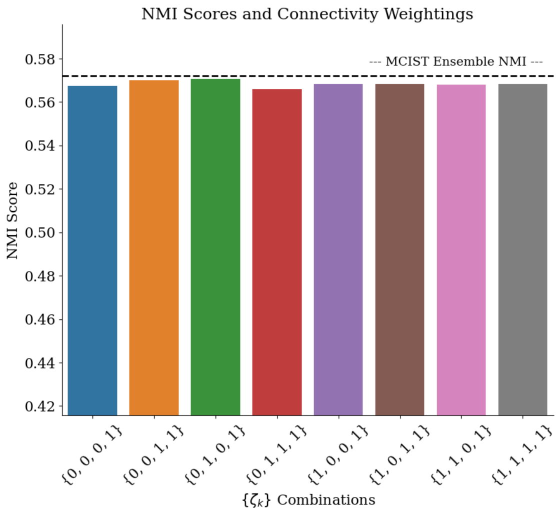

Comparison of NMI scores for different parameter combinations versus NMI score of consensus clustering on DLPFC Slice 151670

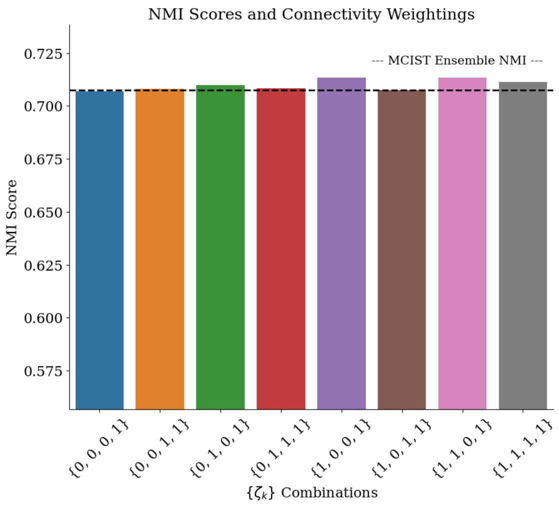

Comparison of NMI scores for different parameter combinations versus NMI score of consensus clustering on DLPFC Slice 151671

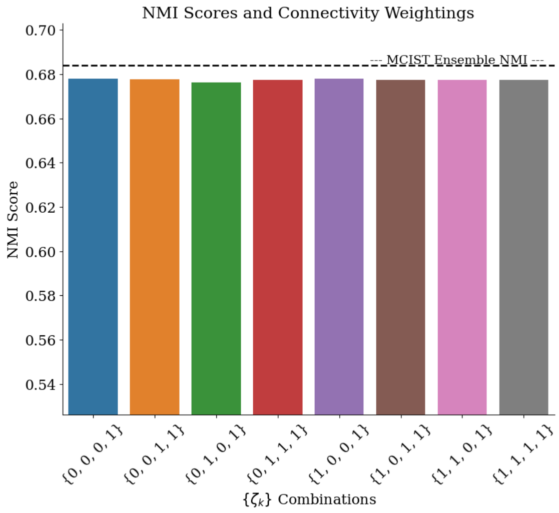

Comparison of NMI scores for different parameter combinations versus NMI score of consensus clustering on DLPFC Slice 151672

Figure S6: NMI Scores for different parameter combinations on DLPFC 151669-151672.

(a) DLPFC Slice 151669

$\{\zeta_k\}$	NMI	RSI
0, 0, 0, 1	0.6299	0.3867
0, 0, 1, 1	0.4992	0.3718
0, 1, 0, 1	0.4985	0.3741
0, 1, 1, 1	0.4945	0.6088
1, 0, 0, 1	0.5985	0.4283
1, 0, 1, 1	0.4959	0.5593
1, 1, 0, 1	0.6141	0.4476
1, 1, 1, 1	0.6307	0.4630

(b) DLPFC Slice 151670

$\{\zeta_k\}$	NMI	RSI
0, 0, 0, 1	0.5674	0.2417
0, 0, 1, 1	0.5701	0.2278
0, 1, 0, 1	0.5707	0.2284
0, 1, 1, 1	0.5658	0.0942
1, 0, 0, 1	0.5681	0.1806
1, 0, 1, 1	0.5684	0.1825
1, 1, 0, 1	0.5680	0.2290
1, 1, 1, 1	0.5682	0.2287

(c) DLPFC Slice 151671

$\{\zeta_k\}$	NMI	RSI
0, 0, 0, 1	0.7068	0.0886
0, 0, 1, 1	0.7080	0.1181
0, 1, 0, 1	0.7099	0.1206
0, 1, 1, 1	0.7084	0.1415
1, 0, 0, 1	0.7135	0.1396
1, 0, 1, 1	0.7075	0.1335
1, 1, 0, 1	0.7134	0.1366
1, 1, 1, 1	0.7113	0.1768

(d) DLPFC Slice 151672

$\{\zeta_k\}$	NMI	RSI
0, 0, 0, 1	0.6779	0.2675
0, 0, 1, 1	0.6777	0.2972
0, 1, 0, 1	0.6762	0.3135
0, 1, 1, 1	0.6774	0.0563
1, 0, 0, 1	0.6780	0.1070
1, 0, 1, 1	0.6775	0.1388
1, 1, 0, 1	0.6775	0.0006
1, 1, 1, 1	0.6775	0.3308

Table S16: NMI and RSI Scores by ζ Combinations on DLPFC Slices 151669, 151670, 151671, and 151672

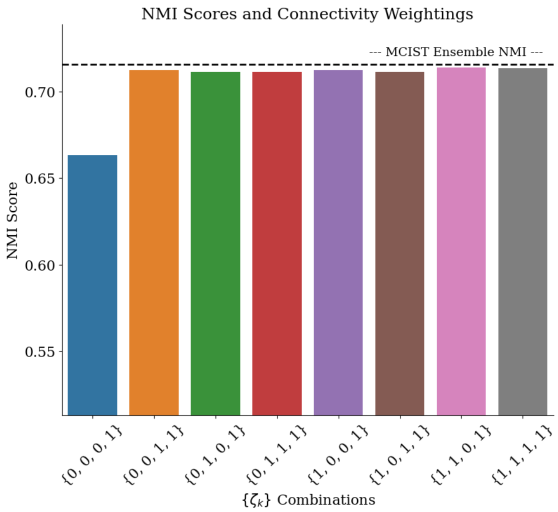

Comparison of NMI scores for different parameter combinations versus NMI score of consensus clustering on DLPFC Slice 151673

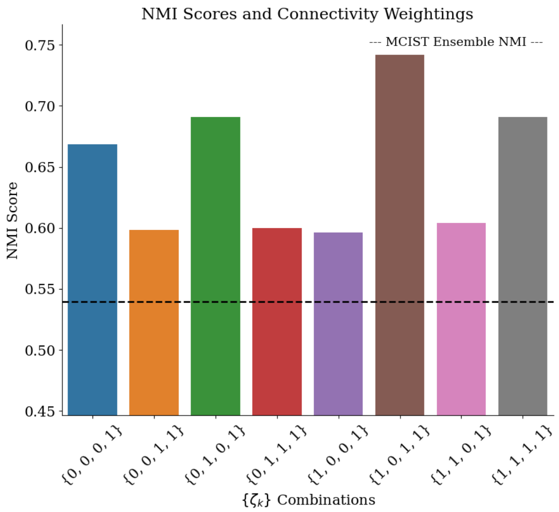

Comparison of NMI scores for different parameter combinations versus NMI score of consensus clustering on DLPFC Slice 151674

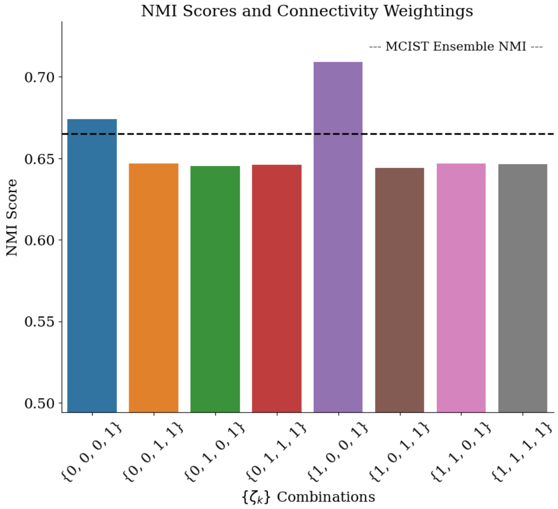

Comparison of NMI scores for different parameter combinations versus NMI score of consensus clustering on DLPFC Slice 151675

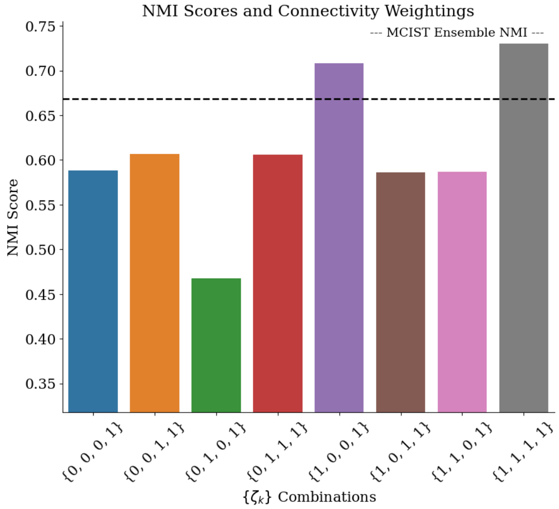

Comparison of NMI scores for different parameter combinations versus NMI score of consensus clustering on DLPFC Slice 151676

Figure S7: NMI Scores for different parameter combinations on DLPFC 151673-151676.

(a) DLPFC Slice 151673

$\{\zeta_k\}$	NMI	RSI
0, 0, 0, 1	0.6632	0.1354
0, 0, 1, 1	0.7124	0.2212
0, 1, 0, 1	0.7114	0.1763
0, 1, 1, 1	0.7113	0.2473
1, 0, 0, 1	0.7122	0.1612
1, 0, 1, 1	0.7114	0.2343
1, 1, 0, 1	0.7138	0.2125
1, 1, 1, 1	0.7135	0.2290

(b) DLPFC Slice 151674

$\{\zeta_k\}$	NMI	RSI
0, 0, 0, 1	0.6684	0.3851
0, 0, 1, 1	0.5982	0.4271
0, 1, 0, 1	0.6908	0.3684
0, 1, 1, 1	0.5999	0.4517
1, 0, 0, 1	0.5962	0.3955
1, 0, 1, 1	0.7418	0.4014
1, 1, 0, 1	0.6040	0.4163
1, 1, 1, 1	0.6906	0.4170

(c) DLPFC Slice 151675

$\{\zeta_k\}$	NMI	RSI
0, 0, 0, 1	0.6741	0.0339
0, 0, 1, 1	0.6470	0.0913
0, 1, 0, 1	0.6451	0.0694
0, 1, 1, 1	0.6462	0.1256
1, 0, 0, 1	0.7090	0.1081
1, 0, 1, 1	0.6441	0.1231
1, 1, 0, 1	0.6469	0.1092
1, 1, 1, 1	0.6464	0.1174

(d) DLPFC Slice 151676

$\{\zeta_k\}$	NMI	RSI
0, 0, 0, 1	0.5885	0.3426
0, 0, 1, 1	0.6066	0.4643
0, 1, 0, 1	0.4678	0.4387
0, 1, 1, 1	0.6057	0.4775
1, 0, 0, 1	0.7080	0.3449
1, 0, 1, 1	0.5857	0.4709
1, 1, 0, 1	0.5867	0.4695
1, 1, 1, 1	0.7300	0.4096

Table S17: NMI and RSI Scores by ζ Combinations on DLPFC Slices 151673, 151674, 151675, and 151676

S4 Residue-Similarity Index Analysis

Given the general lack of availability of ground truth annotations in ST data, we require additional metrics that assess clustering quality without the need for a ground truth. Scores such as Silhouette Score and LISI have been previously proposed, but are not commonly utilized in the literature. Furthermore, we would desire such a metric to also correlate with measures of accuracy and clustering convergence. We have previously proposed the Residue-Similarity Index and R-S plots for visualization of high dimensional single cell RNA-seq data, and have used it to assess clustering performance. The RS-Index is defined as follows [8].

An R-S plot consists of two main components: the residue score and the similarity score. The residue score is calculated as the sum of distances between classes, capturing the dissimilarity between them. On the other hand, the similarity score represents the average similarity within each class, indicating the degree of similarity between instances belonging to the same class. By considering both scores, R-S plots provide a comprehensive representation of the data's structure in a visualization.

Given data of the form $\{(\vec{x}_i, y_i) | \vec{x}_i \in \mathbb{R}^N, y_i \in \mathbb{Z}_l\}_{i=1}^M$, we have y_i representing the class label of our ith data point $\vec{x}_i \in \mathbf{X}$. Say that our data has N samples, M features, and L classes. We can then partition our dataset $\mathbf{X}$ into subsets containing each of the classes by taking $\mathcal{C}_l = \{\vec{x}_i \in \mathbf{X} | y_i = l\}$. For each class l we then define the residue score as follows:

$$R_i := R(\vec{x}_i) = \frac{1}{R_{\max}} \sum_{\vec{x}_j \notin \mathcal{C}_l} \|\vec{x}_i - \vec{x}_j\|, \tag{2}$$

where $\|\cdot\|$ denotes the Euclidean distance between vectors and $R_{\max}$ is the maximal residue score for that subset. The similarity score, meanwhile, is given as:

$$S_i := S(\vec{x}_i) = \frac{1}{|\mathcal{C}_l|} \sum_{\vec{x}_j \in \mathcal{C}_l} \left(1 - \frac{\|\vec{x}_i - \vec{x}_j\|}{d_{\max}}\right), \tag{3}$$

where $d_{\max}$ is the maximal pairwise distance of the dataset. For constructing R-S plots, we then take $R(\vec{x})$ to be the x-axis and $S(\vec{x})$ to be the y-axis.

Class residue index (CRI) and class similarity index (CSI) can be easily defined for the l'th class as $CRI_l = 1/|C_l| \sum_m R_m$ and $CSI_l = 1/|C_l| \sum_m S_m$, respectively. Such indexes can be used to compare the distributions in different classes or clusters obtained by different methods. we then can define the residue index (RI) and similarity index (SI) as $RI = 1/L \sum_l CRI_l$ and $SI = 1/L \sum_l CSI_l$, respectively. The R-S disparity score is then calculated as $RI - SI$ which yields the R-S Index: $1 - |RI - SI|$.

The Rand index is known to correlate with accuracy [8]. We have previously speculated that the R-S disparity may correlate with the convergence of clustering and the R-S index may correlate with the accuracy of classification. R-S disparity and R-S index can be used to measure the performance of different methods. Given that the calculation of the R-S Index is not reliant on any ground truth labels for our data, it would be beneficial to determine how tuning a model to maximize this score can in turn lead to improved accuracy. For our data we observe the following relations in Figure S10, where we have tested different embedding dimensionality values for Topological PCA and noted the relative performances in ARI and RSI. To facilitate a more meaningful comparison of the two metrics, we scaled each of the scores by dataset via MinMax scaling. We then note a 0.60 correlation coefficient on average between RS-Index and ARI on several DLPFC datasets, and a 0.43 correlation coefficient on average for the MERFISH data. Both results are statistically significant with p-values of 0.0002 and 0.03 respectively.

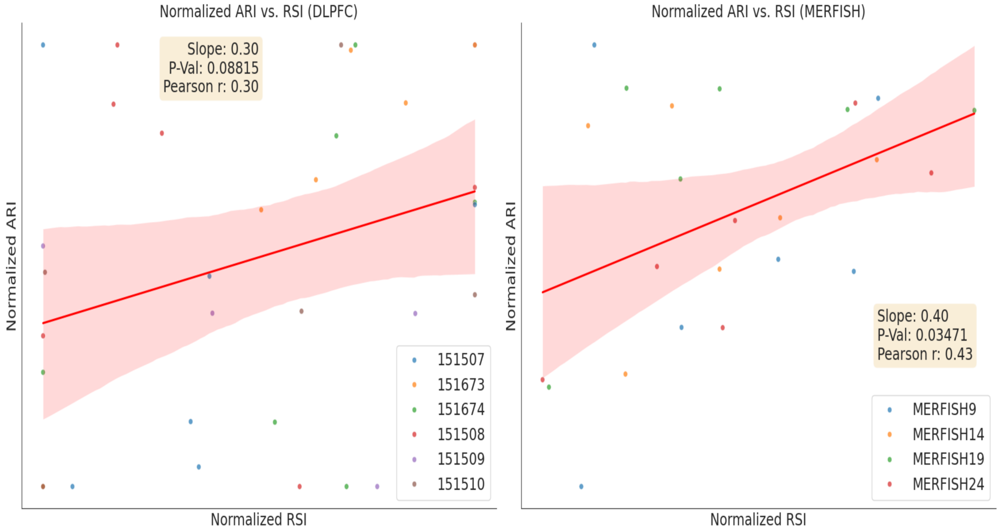

Figure S8: The linear relationship observed between RSI and ARI over several of the DLPFC and MERFISH datasets. RSI and ARI scores are generated by varying the embedding dimensionality parameter, m, while fixing the other parameters.

By focusing on each individual dataset we can assess how the scores vary together as the parameter varies. Again the RSI and ARI scores are normalized such that they can be directly compared. We observe in Figure S9 that the metrics tend to move together in general, illustrating the utility of utilizing the R-S Index as an objective metric for fine-tuning a model, especially in the absence of ground truth labels.

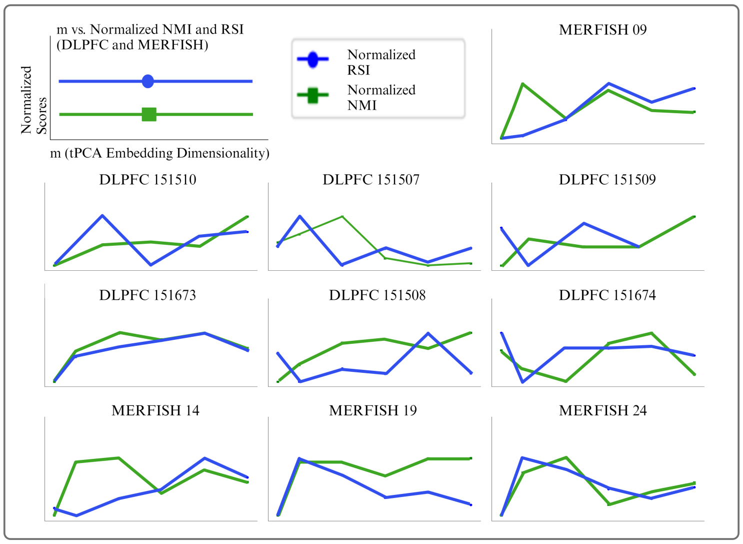

Figure S9: Relationships observed between normalized RSI and ARI scores over individual datasets as the m parameter varies.

We observe similar trends for Normalized Mutual Information in Figure S8, this time with a 0.53 correlation coefficient on average for the MERFISH data and 0.57 for the DLPFC data. Both results are again statistically significant with p-values of 0.007 and 0.0005 respectively.

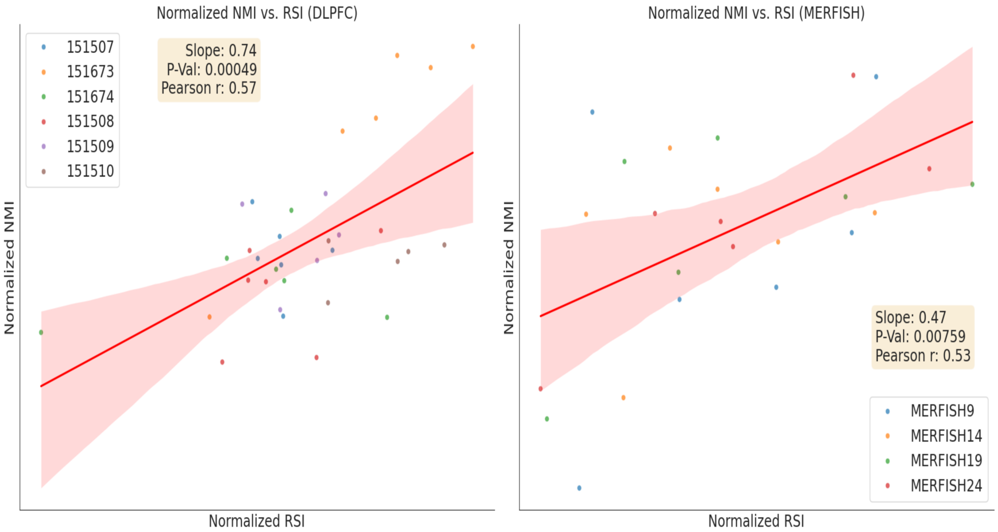

Figure S10: The linear relationship observed between normalized RSI and NMI over several of the DLPFC and MERFISH datasets. RSI and NMI scores are generated by varying the embedding dimensionality parameter, m, while fixing the other parameters.

S5 Filtration Overview

In Figure S11, we provide a visual overview of the filtration process, which enables the multi-scale analysis in Topological PCA. We construct a sequence of cell-cell interaction graphs via kNN-induced filtration as described in the main paper, and construct an Accumulated Spectral Graph by computing a weighted sum over the L_0 Laplacians of each sub-graph.

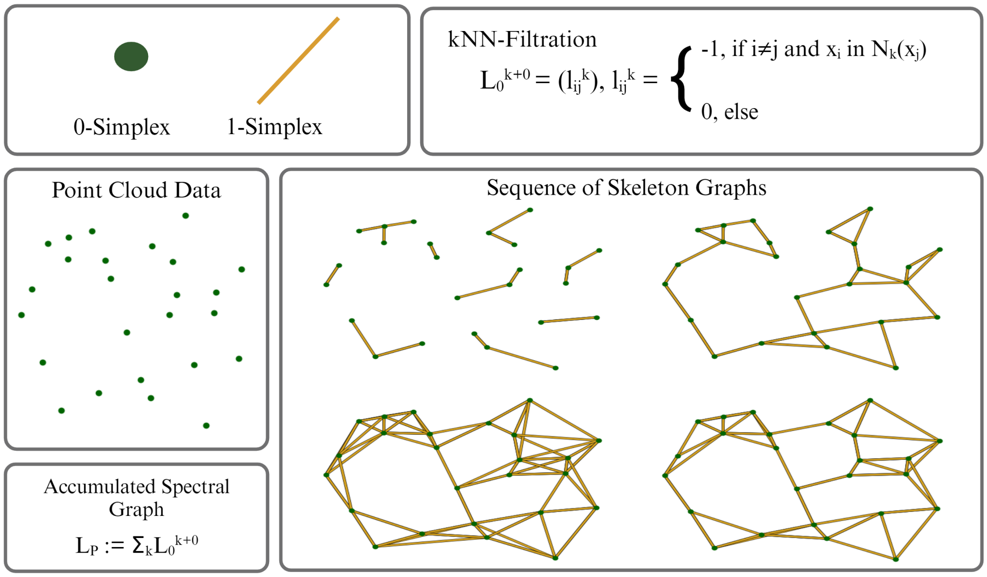

Figure S11: An overview of the filtration procedure utilized in kNN-Topological PCA to induce a multi-scale view of the data. The gene expressin data is treated as a point cloud structure, from which we conST [9]ruct k-Nearest-Neighbors cell-cell similarity graphs, where we vary the k parameter to generate a sequence of skeleton graphs. The graph Laplacian matrices of each graph structure are summed over to produce the Accumulated Spectral Graph.

S6 Evaluating MCIST on Tumor Heterogeneity in Visium Breast Cancer Data

In this section we provide an additional study validating the use of MCIST for gaining biological insights from its produced embeddings. Spatial transcriptomics has the advantage over single cell RNA-sequencing in that it can preserve the spatial context of gene expression within tissues, which is crucial for understanding the tumor microenvironment. Intratumoral heterogeneity in cancer can greatly complicate effective approaches to treatment, significantly hurting survival prospects. In this analysis, we performed clustering on a 10x Visium human breast cancer dataset with 3,798 spots and 36,601 genes. The data was manually annotated based on the H&E image and the spatial expression of reported breast cancer marker genes. The data was first annotated as four regions: healthy tissue, non-invasive, invasive cancer regions, and surrounding tumor regions. These annotations were then refined to twenty and grouped into four main morphotypes: DCIS/LCIS, IDC, Tumor edges, and healthy tissue. In Figure S12, for a simple comparison we performed clustering with five different methods and can visually assess that all seem to visually agree with the manual annotations, with the SEDR [10] and Scanpy clusters being slightly more fragmented and discontinuous.

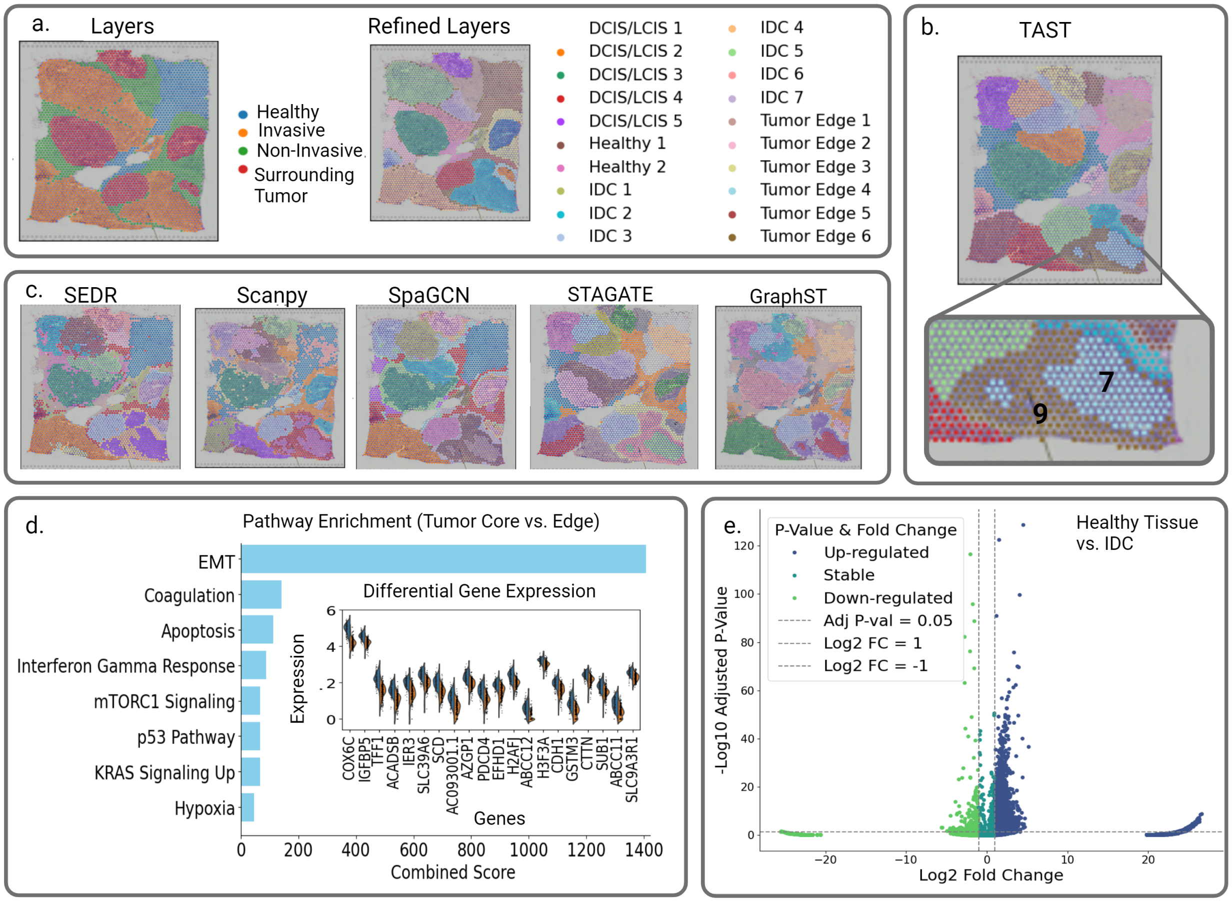

Figure S12: a. Ground truth annotations of Visium Breast tumor data. Regions are annotated as healthy and cancerous regions and then labels are refined to specific cancer regions to reflect the tumor micro-environment. b. MCIST spatial domains are able to accurately indicate the tumor micro environment. We specifically highlight a tumor edge and core region for further analysis. c. Comparisons of spatial domains of other notable methods. Certain methods, specifically Scanpy, detect very fragmented and discontinuous domains. d. Differential gene expression and pathway enrichment analysis between tumor core and edge exposes certain biologically significant differences between different regions of the tumor micro environment. e. Volcano plot of differentially expressed genes between the healthy tissue and IDC regions highlights statistically significant up-regulated genes in the invasive cancer regions.

We note that each method, particularly STAGATE [11] and MCIST when paired with STAGATE, divided the tumor region in the lower right section of the sample into a core and edge structure, clusters 7.0 and 9.0 respectively in the MCIST spatial domains. We again performed differential expression and pathway enrichment analysis between the two regions. Interestingly, we observe significant variance between the clusters for epithelial mesenchymal transition, which we might expect to be occuring more at the tumor edge as this is the invasive front of the tumor [12]. Also variance among interferon gamma response and antigen processing, which allows the immune system to recognize tumor cells, could occur due to the tumor edge being closer to the tissue's immune cells [13]. The tumor core is also significantly hypoxic, and we would expect there to be changes in gene expression between the core and tumor edge to promote survival in low oxygen conditions [14]. In this analysis we have thus shown that MCIST can detect intratumoral heterogeneity within visually homogeneous tumor regions, and its embeddings can be used to further understand the biological processes which differ between different tumor micro-environments.

S7 Evaluating MCIST on Neurodevelopment Trajectory Inference

As noted in Section 2.3 of the main text, newly born neurons form in the ventricular zone and migrate through the intermediate zone along radial glia fibers to the cortical plate, which develops between the marginal zone and subplate. The cortical plate forms in an inside-out fashion, meaning that our pseudotime values should increase in a smooth, continuous fashion from the innermost neuronal layers / White Matter out towards the the outermost layers.

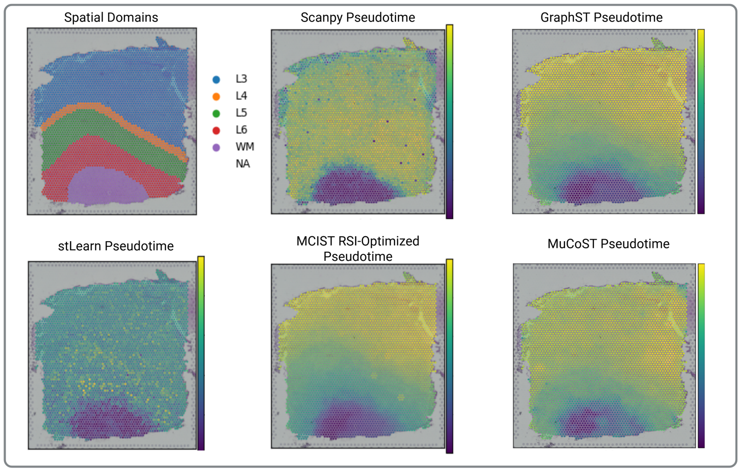

Figure S13: Pseudotime values on slice #151671 of the Visium DLPFC data. Results demonstrate that the MCIST embeddings are able to accurately exhibit the "inside-out" pattern of neurodevelopment while other popular methods, such as Scanpy and stLearn [15], struggle to do so in a smooth or clear fashion.

In Figure S13, for Scanpy, we observe a lack of layered patterns as well as significant noise. This is unsurprising given that Scanpy is not a spatial method. Meanwhile, stLearn [15] is a spatially resolved technique, yet still struggled to form continuous layers or a smooth gradient when we performed trajectory inference on the morhpology regularized PCA embeddings. Furthermore, neither method seems able to clearly depict the desired inside out pattern. MCIST, however, exhibits a clear, layer-patterned gradient extending smoothly out towards the outskirts of the cortex, accurately reflecting the desired temporal orderings. This further validates the usefulness of MCIST in performing meaningful downstream analysis after spatial domain detection, including in instances where other popular methods struggle. Additionally, two other spatially resolved deep learning methods, MuCoST and GraphST, manage to provide a fairly smooth gradient extending outward from the innermost white matter region. However, the MuCoST pseudotime values find difficulty in distinguishing the temporal ordering of the white matter and inner layer regions-particularly layers 6 and 5. The GraphST pseudotime values generally align with the MCIST values, with the exception of MCIST providing a relatively clearer gradient between layers 4 and 3, where the transition from 'inner' to 'outter' layers occurs.

S8 Evaluation Metrics

S8.1 Adjusted Rand Index (ARI)

ARI describes how well two clusterings agree with each other by comparing pairs of data points and their respective class assignments. It also can account for possible random agreement and adjusts the similarity score accordingly, assigning a value in the range of -1 to 1. A value of 1 indicates a perfect agreement between clusterings, a value of 0 indicates a random chance agreement, and a value of -1 suggests that the clusterings

are less similar than they would be by chance. For two clusterings $X = \{X_1, ..., X_r\}$ and $Y = \{Y_1, ..., Y_s\}$, we conST [9]ruct a contingency table $A \in \mathbb{R}^{r \times s}$ with elements a_{ij} which describe the overlap between X_i and Y_j. We then take row sums and column sums to obtain another set of values: $\{q_1, ..., q_r\}$ is the set of row sums and $\{p_1, ..., p_s\}$ is the set of column sums. We can then define the Adjusted Rand Index as:

$$\text{ARI} = \frac{\sum_{ij} \binom{a_{ij}}{2} - (\sum_i \binom{q_i}{2} \sum_j \binom{p_j}{2})}{\frac{1}{2}(\sum_i \binom{q_i}{2} + \sum_j \binom{p_j}{2}) - (\sum_i \binom{q_i}{2} \sum_j \binom{p_j}{2})} \tag{4}$$

S8.2 Normalized Mutual Information (NMI)

Mutual Information considers a split of the data according to clusters and a split according to true class labels, and measures how these splittings agree with each other. NMI then corrects for any bias and normalizes the scores between 0 and 1. A value of 1 indicates a perfect agreement between the splittings while a value of 0 indicates random chance agreement. The mathematical definition of NMI is given as:

$$\text{NMI}(T, P) = \frac{\text{MI}(T, P)}{(\text{E}(T) + \text{E}(P))/2} \tag{5}$$

Where $\text{MI}(\cdot, \cdot)$ and $\text{E}(\cdot)$ represent mutual information and entropy and T, P represent the true and predicted cluster labels, respectively.

References

[1] Chenglong Xia, Hazen P Babcock, Jeffrey R Moffitt, and Xiaowei Zhuang. Multiplexed detection of rna using merfish and branched dna amplification. *Scientific reports*, 9(1):7721, 2019.

[2] Alma Andersson, Ludvig Larsson, Linnea Stenbeck, Fredrik Salmén, Anna Ehinger, Sunny Z Wu, Ghamdan Al-Eryani, Daniel Roden, Alex Swarbrick, Åke Borg, et al. Spatial deconvolution of her2-positive breast cancer delineates tumor-associated cell type interactions. *Nature communications*, 12(1):6012, 2021.

[3] Kristen R Maynard, Leonardo Collado-Torres, Lukas M Weber, Cedric Uytingco, Brianna K Barry, Stephen R Williams, Joseph L Catallini, Matthew N Tran, Zachary Besich, Madhavi Tippani, et al. Transcriptome-scale spatial gene expression in the human dorsolateral prefrontal cortex. *Nature neuroscience*, 24(3):425–436, 2021.

[4] Ao Chen, Sha Liao, Mengnan Cheng, Kailong Ma, Liang Wu, Yiwei Lai, Xiaojie Qiu, Jin Yang, Jiangshan Xu, Shijie Hao, et al. Spatiotemporal transcriptomic atlas of mouse organogenesis using dna nanoball-patterned arrays. *Cell*, 185(10):1777–1792, 2022.

[5] Xiaoyin Chen, Yu-Chi Sun, George M Church, Je Hyuk Lee, and Anthony M Zador. Efficient in situ barcode sequencing using padlock probe-based BaristaSeq. *Nucleic Acids Research*, 46(4):e22–e22, 11 2017.

[6] Xiao Wang, William E Allen, Matthew A Wright, Emily L Sylwestrak, Nikolay Samusik, Sam Vesuna, Kathryn Evans, Cindy Liu, Charu Ramakrishnan, Jia Liu, et al. Three-dimensional intact-tissue sequencing of single-cell transcriptional states. *Science*, 361(6400):eaat5691, 2018.

[7] 10x Genomics. Human breast cancer - block a section 1-1 standard 1.0.0. https://www.10xgenomics.com/datasets/human-breast-cancer-block-a-section-1-1-standard-1-0-0, 2023. Accessed: 2024-04-27.

[8] Yuta Hozumi, Rui Wang, and Guo-Wei Wei. Ccp: Correlated clustering and projection for dimensionality reduction. *arXiv preprint arXiv:2206.04189*, 2022.

[9] Yongshuo Zong, Tingyang Yu, Xuesong Wang, Yixuan Wang, Zhihang Hu, and Yu Li. const: an interpretable multi-modal contrastive learning framework for spatial transcriptomics. *BioRxiv*, pages 2022–01, 2022.

[10] Hang Xu, Huazhu Fu, Yahui Long, Kok Siong Ang, Raman Sethi, Kelvin Chong, Mengwei Li, Rom Uddamvathanak, Hong Kai Lee, Jingjing Ling, et al. Unsupervised spatially embedded deep representation of spatial transcriptomics. *Genome Medicine*, 16(1):12, 2024.

[11] Kangning Dong and Shihua Zhang. Deciphering spatial domains from spatially resolved transcriptomics with an adaptive graph attention auto-encoder. *Nature communications*, 13(1):1739, 2022.

[12] Ping Jiang, Atsushi Enomoto, and Masahide Takahashi. Cell biology of the movement of breast cancer cells: intracellular signalling and the actin cytoskeleton. *Cancer letters*, 284(2):122–130, 2009.

[13] Maria Vittoria Dieci, Federica Miglietta, and Valentina Guarneri. Immune infiltrates in breast cancer: recent updates and clinical implications. *Cells*, 10(2):223, 2021.

[14] Gregg L Semenza. The hypoxic tumor microenvironment: A driving force for breast cancer progression. *Biochimica et Biophysica Acta (BBA)-Molecular Cell Research*, 1863(3):382–391, 2016.

[15] Duy Pham, Xiao Tan, Brad Balderson, Jun Xu, Laura F Grice, Sohye Yoon, Emily F Willis, Minh Tran, Pui Yeng Lam, Arti Raghubar, et al. Robust mapping of spatiotemporal trajectories and cell–cell interactions in healthy and diseased tissues. *Nature communications*, 14(1):7739, 2023.
